# Structure–function studies of the C3/C5 epimerases and C4 reductases of the *Campylobacter jejuni* capsular heptose modification pathways

**DOI:** 10.1016/j.jbc.2021.100352

**Published:** 2021-01-30

**Authors:** Heba Barnawi, Laura Woodward, Natalie Fava, Mikhail Roubakha, Steve D. Shaw, Chelsea Kubinec, James H. Naismith, Carole Creuzenet

**Affiliations:** 1 Department of Microbiology and Immunology, University of Western Ontario, London, Ontario, Canada; 2 Biomedical Sciences Research Complex, St Andrews University, St Andrews, UK; 3 Rosalind Franklin Institute, Research Complex at Harwell, Harwell Campus, Didcot, UK; 4 Division of Structural Biology, Oxford University, Oxford, UK

**Keywords:** *Campylobacter jejuni*, capsule, heptose, epimerase, reductase, CE, capillary electrophoresis, EvaD, dTDP-4-keto-6-deoxy-d-glucose-5-epimerase, GFS, GDP-L-fucose synthase, GME, GDP-mannose C3/C5 epimerase, GMER, GDP-4-keto-6-deoxy-D-mannose C3/C5 epimerase, SDR, short chain dehydrogenase reductase, SEC-MALS, size-exclusion chromatography coupled with multiangle light scattering

## Abstract

Many bacteria produce polysaccharide-based capsules that protect them from environmental insults and play a role in virulence, host invasion, and other functions. Understanding how the polysaccharide components are synthesized could provide new means to combat bacterial infections. We have previously characterized two pairs of homologous enzymes involved in the biosynthesis of capsular sugar precursors GDP-6-deoxy-D-*altro*-heptose and GDP-6-OMe-L-*gluco*-heptose in *Campylobacter jejuni*. However, the substrate specificity and mechanism of action of these enzymes—C3 and/or C5 epimerases DdahB and MlghB and C4 reductases DdahC and MlghC—are unknown. Here, we demonstrate that these enzymes are highly specific for heptose substrates, using mannose substrates inefficiently with the exception of MlghB. We show that DdahB and MlghB feature a jellyroll fold typical of cupins, which possess a range of activities including epimerizations, GDP occupying a similar position as in cupins. DdahC and MlghC contain a Rossman fold, a catalytic triad, and a small C-terminal domain typical of short-chain dehydratase reductase enzymes. Integrating structural information with site-directed mutagenesis allowed us to identify features unique to each enzyme and provide mechanistic insight. In the epimerases, mutagenesis of H67, D173, N121, Y134, and Y132 suggested the presence of alternative catalytic residues. We showed that the reductases could reduce GDP-4-keto-6-deoxy-mannulose without prior epimerization although DdahC preferred the pre-epimerized substrate and identified T110 and H180 as important for substrate specificity and catalytic efficacy. This information can be exploited to identify inhibitors for therapeutic applications or to tailor these enzymes to synthesize novel sugars useful as glycobiology tools.

Bacteria produce sugars often found as part of or attached to the cell wall or in a capsule where they are often important for virulence and in some cases cell viability. We have previously characterized the dehydratases, epimerases, and reductases that modify GDP-*manno*-heptose into various heptose forms found in the capsule of *Campylobacter jejuni* (Fig. 1) and in the lipopolysaccharide of *Yersinia pseudotuberculosis* (1, 2, 3, 4) (not shown). Specifically, the *Y. pseudotuberculosis* pathway leading to 6-deoxy-D-*manno*-heptose was a simple two-step pathway consisting of C4, C6 dehydration, and C4 reduction of GDP-*manno*-heptose (1). In contrast, the *C. jejuni* Ddah and Mlgh modification pathways leading to 6-deoxy-D-*altro*-heptose and 3,6-OMe-L-*gluco* heptose, respectively, have been found to be more complex (Fig. 1). Both pathways operate *via* a 6-deoxy-4-keto intermediate (P1 formed by DdahA (aka WcbK) (2)), which is then processed by a pair of enzymes (DdahB/C and MlghB/C). Based on sequence similarity, all enzymes were predicted to be C3/C5 epimerases with DdahC and MlghC possessing additional C4 reductase activity (3, 4). We showed experimentally that DdahB is a C3 epimerase leading to the formation of a single C3 epimerized product (denoted as P4α), whereas MlghB is a C3/C5 epimerase leading to the formation of three products encompassing the same C3 only epimer (P4α) as DdahB but also the C5 only epimer (denoted as P4β) and the double C3, C5 epimer (denoted as P4γ) (Fig. 1). DdahB and MlghB were devoid of reductase activity, but both DdahC and MlghC served as C4 reductases, reducing epimerized products made by DdahB and MlghB to generate products denoted as P5α and P5γ, respectively (3, 4).

**Figure 1:**
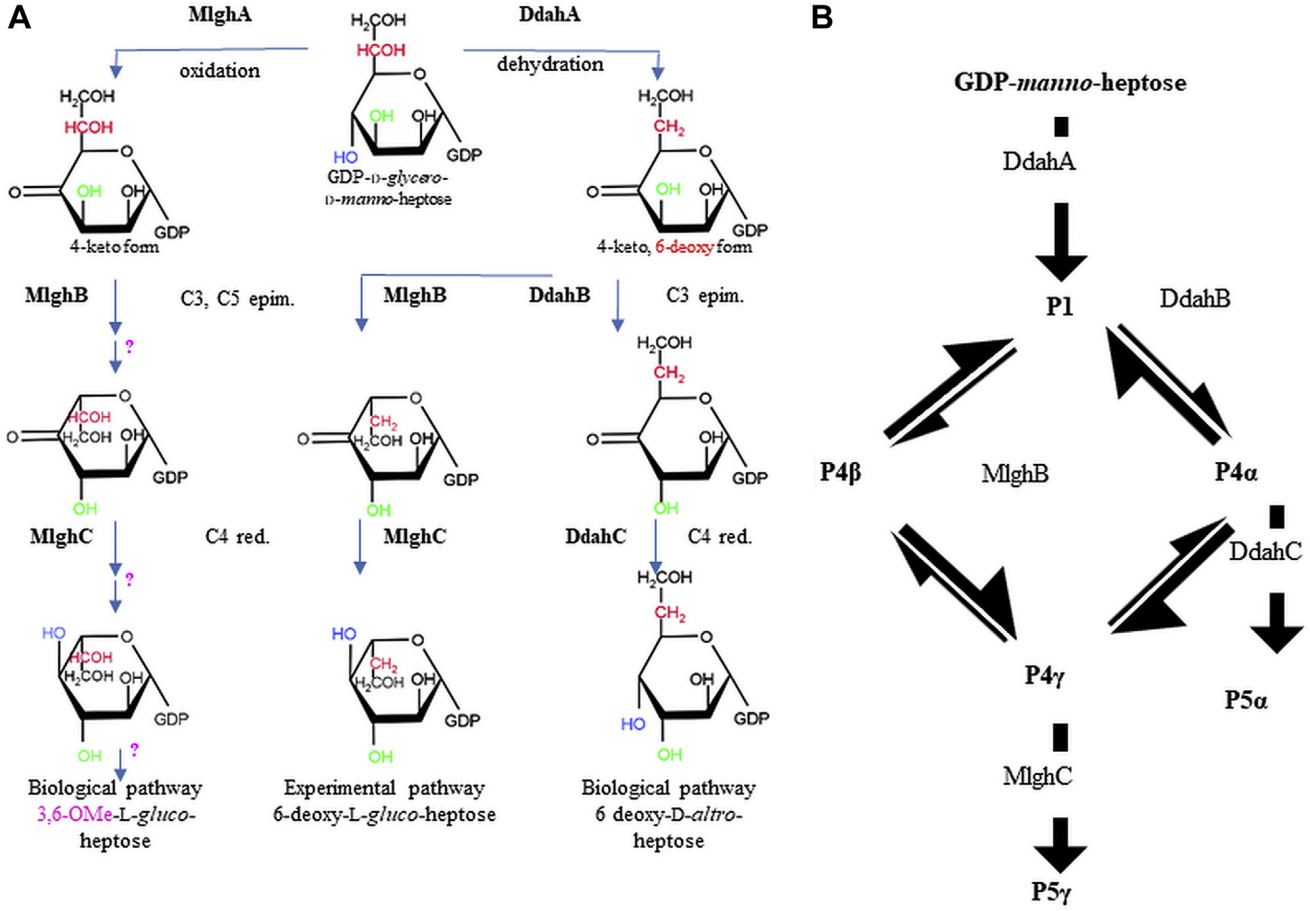
**GDP-*manno*-heptose modification pathways of *Campylobacter jejuni*.***A*, biological pathways leading to 3,6-OMe-L-*gluco*-heptose (*left*) and 6-deoxy-D-*altro*-heptose (*right*). The 3,6-O methylation steps necessary to generate 3,6-OMe-L-*gluco*-heptose have not yet been elucidated and are denoted by ?. Because MlghA had not been identified at onset of our work, an experimental pathway initiated by dehydration by DdahA and leading to 6-deoxy-L-*gluco* heptose (*center*) was used to study MlghB/C and allowed to compare their function directly to DdahB/C. *Red*: reduction. *B*, detailed epimerization and reduction steps showing the three products generated by MlghB (C3 epimer P4α, C5 epimer P4β, and C3/C5 epimer P4γ) *versus* 1 product for DdahB (P4α), as well as the substrate specificity of the 2 reductases MlghC and DdahC. The names of products P1, P4α, P4β, P4γ, P5α, and P5γ are as per McCallum *et al.*, 2013, and are used throughout this article for the sake of clarity and consistency. Epim, epimerization.

In addition, *in vitro* and *in vivo* studies demonstrated that all these enzymes are important for the function of the polysaccharide that incorporates their reaction products, namely contributing to capsule- or lipopolysaccharide-based resistance to serum, bile salts, and/or antibiotics, allowing epithelial cell invasion and playing an essential role in gut colonization and/or dissemination to deeper organs (5, 6, 7). Thus, these enzymes could be novel targets, allowing inhibition of colonization by *Y. pseudotuberculosis* or *C. jejuni* or by other mucosal pathogens that also produce similar modified heptoses, such as *Burkholderia* species (8, 9). This would present alternatives to antibiotics of interest both in human and veterinary medicine (10, 11). *C. jejuni* is of particular concern because it causes severe gastrointestinal disease in humans and its high level of resistance to fluoroquinolones has warranted its classification as a high priority pathogen by the WHO in 2017. With ∼9500 reported cases yearly in Canada (12) (http://publications.gc.ca/pub?id=9.507317&sl=0, https://diseases.canada.ca/notifiable/), campylobacteriosis is often contracted *via* consumption of contaminated undercooked chicken meat. It may be possible to exploit heptose-modifying enzymes as targets to reduce chicken colonization by *C. jejuni* to curtail transmission to humans.

The DdahB/C and MlghB/C pairs of *C. jejuni* enzymes are present in most *Campylobacters* and unique to *Campylobacters* (13, 14), making them attractive targets. Understanding the molecular basis for their specificity would assist in the design of highly selective inhibitors, which would avoid problems of bacterial antibiotic resistance linked to the usage of broad-spectrum antibiotics in chicken farming (10, 11).

The mechanisms involved in C3/C5 epimerization are fairly well understood. There are two main mechanisms extant, the first typified by the dTDP-6-deoxy-D-*xylo*-4-hexulose C3/C5 epimerase RmlC (15, 16, 17). DdahB and MlghB are 38 and 37% identical to *Salmonella enterica* RmlC, respectively. The residues involved in RmlC activity are summarized in Table S1. They include the His 63-Asp 170 dyad, Tyr 133 and Lys 73, which are highly conserved in other bacteria, including *Streptococcus suis* (Table S1 and (17)). The His-Asp dyad is important for both C3 and C5 epimerization of the substrate because RmlC has no cofactor: His 63 acts as a base to deprotonate the sugar from the lower face of the ring at C3 and C5 positions (16) and Asp 170 increases the basicity of His 63, enhancing its ability to deprotonate the sugar. The enolate anion is stabilized by Lys 73 (16, 17). On the opposite face of the sugar ring, Tyr 133 donates a proton at C5 position from its hydroxyl group, thus acting as an acid. A conserved water molecule found close to C3 was proposed to play a role in C3 epimerization (17).

The second mechanism is exhibited by GDP-L-fucose synthase (GFS) (also known as GMER), a GDP-4-keto-6-deoxy-D-mannose C3/C5 epimerase, C4 reductase (18, 19, 20, 21), or GME, a GDP-mannose C3/C5 epimerase from *Arabidopsis thaliana*. These enzymes have an NADP cofactor and carry out four reactions at a single active site: an oxidation, two epimerizations, and a reduction (21, 22). DdahC and MlghC are 44 and 38% identical to *Escherichia coli* GFS, respectively. Residues important for these activities are summarized in Table S2. The reduction reaction is catalyzed by Ser 107, Tyr 136, and Lys 140, which form the typical SYK catalytic triad of short-chain dehydrogenase reductase enzymes (23). Ser 107 and Lys 140 lower the pKa of Tyr 136, which allows it to function as a general acid or base during catalysis through its hydroxyl side chain (19, 21). This residue is important for final reduction of the 4-keto intermediate once epimerization is complete. In GFS, Cys 109 and His 179 form a catalytic dyad where Cys 109 is the base and His 179 is the acid for epimerization at both C3 and C5 positions (19). Functionally equivalent residues are Cys 145/Lys 217 in GME.

Because all known C3/C5 epimerases, C4 reductases, described above use hexoses as substrates while the *Campylobacter* enzymes function in heptose modification pathways, we undertook the investigation of the *Campylobacter* enzymes to decipher the extent of their heptose *versus* mannose specificity and also to understand why all enzymes predicted to be C3/C5 epimerases with C4 reductase activity performed different reactions on different heptose intermediates. The studies involve structural studies of both enzymes, modeling, site-directed mutagenesis, and functional analysis on GDP-*manno*-heptose and GDP-mannose–derived substrates. These studies will support the future applications mentioned above but are also of fundamental importance to provide a better understanding of complex glycan synthesis.

## Results

### Specificity of epimerases MlghB and DdahB

**Figure 2:**
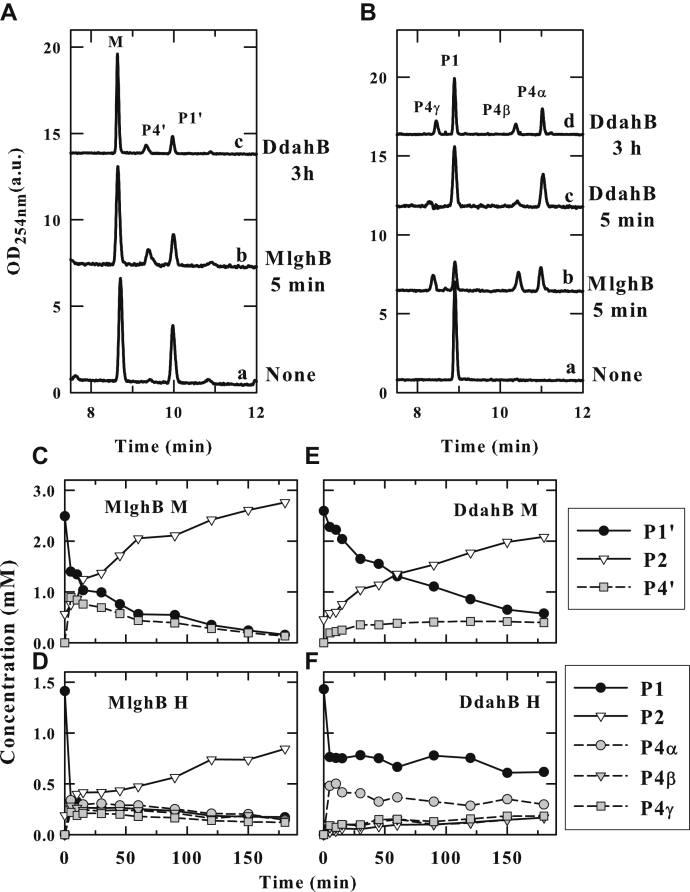
**Specificity of MlghB and DdahB for heptose *versus* mannose.***A* and *B*, capillary electrophoresis (CE) traces of reactions performed by DdahB or MlghB on GDP-6-deoxy-4-keto-mannulose (P1’, *A*) or heptulose (P1, *B*) obtained by incubating GDP-mannose (M) or GDP-*manno*-heptose (H) with DdahA. DdahA was removed by ultrafiltration before the addition of DdahB or MlghB. P4’: new catalysis product arising from mannose-based P1’. For heptose catalysis, all peaks are as described in Figure 1. *C*–*F*, time course of substrate conversion for reactions performed in parallel with heptose (*D* and *F*) *versus* mannose (*C* and *E*) for MlghB (*C* and *D*) or for DdahB (*E* and *F*). The degradation product P2 (*i.e.*, GDP) that forms upon lengthy incubation of P1 and P1’ (migrating at ∼13 min) is accounted for in the quantitation of panels *C*–*F*. The procedure and stoechiometries used to achieve equimolar substrate amounts for all reactions are detailed in Experimental procedures section. This is a representative example of three independent experiments.

MlghB and DdahB are the only C3/C5 epimerases demonstrated to have activity on heptose-based substrates to date. To assess their specificity, GDP-*manno*-heptose and GDP-mannose were converted into the 4-keto, 6-deoxy-derivatives P1 (7 carbon) and P1’ (6 carbon) by DdahA and equimolar amounts were used as substrates for MlghB and DdahB. As established before (3, 4), MlghB converts heptose-based P1 into C3, C5, and C3/C5 epimers (P4α, P4β, and P4γ, respectively) (Figs. 1 and 2, *B* and *D*). In contrast, DdahB mostly converts the P1 substrate to P4α (C3 epimer) (Fig. 2, *B* and *F*), but prolonged incubation (3 h) with DdahB led to appearance of P4β (C5 only) and P4γ (C3 and C5) (Fig. 2*B*) and concomitant decrease in the P4α peak. Because there was no further conversion of P1 in that time frame, this suggests that C5 epimerization also occurred, generating P4β and further converting P4α into P4γ.

Both enzymes were able to epimerize a mannose (six carbon) derived substrate denoted P1’ into product P4’ (the nomenclature is chosen to mirror the heptose conversion with the addition of the prime denoting the six-carbon substrate). P1’ was processed much less efficiently than P1, especially by DdahB where catalysis was limited even after a 3-h incubation and was accompanied by significant substrate degradation (Fig. 2, *A*, *C* and *E*). DdahB thus possesses clear “heptose preference.” Definitely establishing the nature of P4’ has proven impossible because of its instability and of the very small amounts of material produced. Because DdahB primarily catalyzes C3 epimerization activity of heptose (3, 4), we reasoned that P4’ would be the C3 epimer. This would imply MlghB was incapable of C5 epimerization of the smaller mannose-based substrate. The additional carbon of the P1 substrate is critical for enzyme turnover, appearing essential for the C5 position.

### Structural studies of epimerases MlghB and DdahB

**Figure 3:**
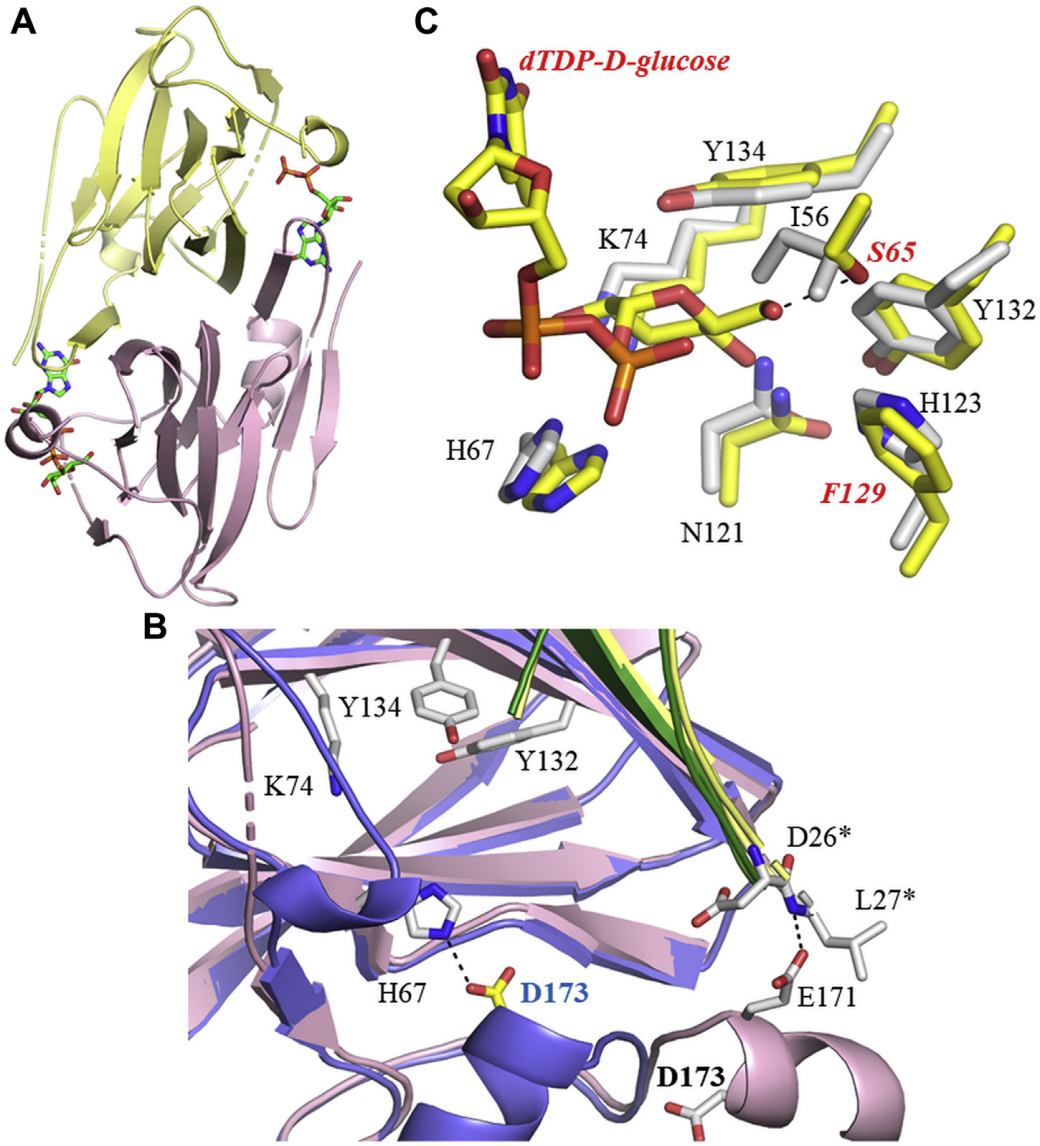
**Structural data on DdahB and MlghB.***A*, the dimer of DdahB is shown as a *cartoon*, with one monomer in *yellow* and the other in *pink*. GDP and GDP-mannose are shown as *sticks* with carbons *green*, oxygen *red*, nitrogen *blue*, and phosphorous *orange*. *B*, close-up view of the superposition of the DdahB dimer (colored as in panel *A*) with MlghB (shown in *green* and *blue*). Residues shown in *sticks* are colored with carbons in *white* for DdahB and in *yellow* for MlghB. Other atoms are colored as in panel *A*. Residues numbered with ∗ belong to the second subunit of the dimer. Residues highlighted are those whose role was tested experimentally by site-directed mutagenesis: K74, Y134, Y132 H67, and D173. Asp 173 (shown in *bold black* for DdahB and *blue* for MlghB), a key catalytic residue, is out of position in DdahB because of Glu 171 makes hydrogen bond with the other monomer. *C*, catalytic residues of DdahB monomer are superimposed with *Streptococcus suis* RmlC (1nyw). The side-chain carbon atoms of DdahB are colored *white*, whereas carbon atoms of RmlC are colored *yellow*. The active sites are almost identical, and important residues in DdahB are labeled. Among them, H67, K74 Y134 Y132, and N121 were tested functionally by site-directed mutagenesis. O6 of dTDP-glucose substrate in RmlC hydrogen bonds to Ser 65 and Phe 129 is remote from O6. In DdahB, these residues are found as Ile 56 and His 123. This arrangement is consistent with the larger C7 substrate with C7 making a van der Waal interaction with Ile56 and the hydroxyl on C7 hydrogen bonding to His123.

DdahB and MlghB were purified as homodimers (Fig S1, *A* and *B*, Table S3), crystallized, and their structures solved to 1.30 Å and 2.14 Å resolution, respectively. DdahB crystallized in space group *P*1 2_1_ 1, with two chains in the asymmetric unit and MlghB crystallized in space group *P*2_1_ 2_1_ 2_1_, with four chains in the asymmetric unit. Statistics on the final structures are reported in Table 1. The final structure of DdahB has two monomers in the asymmetric unit that are expected to form a stable dimer (24), consistent with size-exclusion chromatography coupled with multiangle light scattering (SEC-MALS, Fig. 3*A* and Fig. S1*A*). Residues 1 to 139 and 145 to 174 of monomer A and residues 3 to 181 of monomer B are experimentally located. The monomer comprises 14 β-strands and two short α-helices, with nine β-strands, arranged in two β-sheets, forming a small antiparallel β-barrel, known as a jellyroll. The fold of the protein identifies it as a member of the cupin family (25). MlghB has the same cupin fold as DdahB, and the monomers superimpose with an RMSD of 0.7 Å for the 163 overlapping Cα atoms and share 81% sequence identity (Table S4). Like DdahB, MlghB has two dimers (24) in the asymmetric unit (Fig. 3*B*); residues 2 to 178, 3 to 142 and 148 to 178; 3 to 138 and 147 to 173, and 2 to 140 are located in subunits A, B, C, and D, respectively. In the dimer, a β-strand from one monomer adds, in an antiparallel manner, to the β-sheet of the other monomer. The arrangement buries around 20% of the surface area of each monomer. No cofactor was detected in either structure.

**Table 1:** Additional crystallographic parameters for DdahB and MlghB

Parameter measured	DdahB	MlghB	DdahB GDP-mannose	MlghB GDP-mannose
Data collection				
Space group	P2_1_	P2_1_2_1_2_1_	P2_1_	P2_1_2_1_2_1_
Wavelength	0.916	0.916	1.54	1.54
Unit cell dimensions				
a, b, c (Å)	47.8, 68.3, 53.7	47.3, 121.7, 153.8	48.0, 67.8, 53.1	42.4, 121.9, 154.1
α, β, γ (°)	90, 91.4, 90	90, 90, 90	90, 91.8, 90	90, 90, 90
Resolution (Å)^a^	34–1.3 (1.32–1.30)	95–2.14 (2.20–2.14)	53–2.35 (2.43–2.35)	56.7–2.60 (2.67–2.60)
R_merge_^a^	0.047 (0.57)	0.06 (0.76)	0.061 (0.169)	0.162 (0.669)
I/σI^a^	13.9 (1.2)	12.7 (1.1)	16.3 (6.1)	12.5 (3.5)
CC_1/2_^a^	1.0 (0.7)	1.0 (0.5)	1 (0.9)	1.0 (0.8)
Completeness^a^	95 (73)	100 (100)	98 (89)	99 (92)
Redundancy^a^	2.7 (1.4)	5.9 (4.2)	3.6 (2.7)	7.0 (6.8)
Refinement				
Resolution (Å)^a^	34–1.3 (1.32–1.30)	95–2.1 (2.18–2.14)	39–2.35 (2.43–2.35)	57–2.60 (2.67–2.60)
Reflections^a^	76,397 (4400)	41,762 (2944)	13,466 (879)	23,972 (1611)
R_work_/R_free_ %^a^	14.8/17.5 (31.7/30.1)	21.6/23.9 (42.1/42.4)	18.5/24.3 (19.4/31.5)	21.0/23.5 (29.4/34.3)
No. of atoms				
Protein	2742	5648	2742	5858
Water	46	67	46	8
Ligands	-	-	67	112
Residual B factors (Å^2^)				
Protein	21	64	22	2326
Water	48	46	11	16
Ligand	-	-	45	47
RMSD				
Bond lengths (Å)	0.013	0.010	0.007	0.010
Bond angles (°)	1.6	1.4	1.3	1.6
Ramachandran				
Favored (%)	98	99	98	99
Outliers (%)	0	0	0	0
	7ANI	7ANG	7ANJ	7AN4

a Values in parenthesis are for the highest shell.

The fold of the monomer and dimeric arrangement of both DdahB and MlghB closely resemble that of RmlC, the third enzyme in the dTDP-L-rhamnose pathway (Fig. 3*C* and Table S4 for RMSD values). RmlC was the first cupin C3/C5 epimerase enzyme structure to be described (15). The structural similarity is much higher than sequence similarity (38% identity). A search of the RCSB reveals many other cupin fold enzymes including multiple C3/C5 epimerases.

As DdahB and MlghB can both epimerize GDP-mannose (in addition to their normal heptose-based substrates), we attempted to obtain cocomplexes with GDP-mannose. The structure of DdahB in the presence of GDP-mannose was solved to a resolution of 2.35 Å. Although the crystal was soaked in a solution of GDP-mannose prepared in mother liquor before freezing, the electron density was poor. We positioned GDP-mannose in one chain and GDP only in the other (Fig. 3*A* and Fig. S2). The presence of GDP-mannose in one chain suggested to us the problem was not degradation of GDP-mannose but rather the disorder. We attributed this to the fact GDP-mannose is not the true natural substrate and therefore its binding may be suboptimal. There was little change in the protein structure upon substrate binding (Table S4).

MlghB in the presence of GDP-mannose crystallized in the same unit cell as native, diffracted to 2.6 Å, showed little change in structure, but we were only able to locate the GDP moiety for each monomer (Fig. S2 and Table S4). In both proteins, the guanine, ribose, and the phosphate attached to ribose portions of the GDP were bound at the same location, at the dimer interface. In both structures, the guanine ring makes hydrogen bonds to Asn 22, Thr 33, and Lys 54∗ (∗ denotes this residue is located in the other monomer in the dimer), with Ile 3 and Ile 31 stacking on opposite sides of the aromatic ring. The ribose ring interacts with the side chain of Phe 24. In MlghB, Asp 144∗ is hydrogen bonded to the ribose O2 atom, but the equivalent loop is disordered in DdahB. The phosphate group makes a salt bridge to Arg 28 in DdahB in the subunit where GDP-mannose has been positioned. In the subunit where only GDP is in position in addition to Arg 28, there is a salt contact to Arg 172. In MlghB, this phosphate makes a salt link to Arg 64∗ and Arg 172∗.

Comparison to RmlC from *St. suis* in complex with dTDP-glucose (16) has revealed that the location of the thymidine, ribose, and attached phosphate is essentially in the same with respect to the structure. Although there are differences in detail of the specific molecular interactions, the location of the residues and the type of contacts that they form (hydrogen bond, van der Waals, salt bridge) are conserved. The phosphate attached to the ribose ring in RmlC contacts Arg 33 and Arg 73, equivalent to Arg 28 and Arg 64∗ in DdahB and MlghB. In MlghB, the β-phosphate did not make either hydrogen or salt bridges with the residues from either monomer. The positioning of the β-phosphate differs between subunits in DdahB. Where GDP-mannose was placed in density, the β-phosphate is on the surface of the protein where it contacts His 67. In the other subunit with only GDP placed, the phosphate adopts a different position and points into a cavity to make salt contacts with Arg 28, Arg 64∗, and His 67∗. Superposition of the four monomers in the asymmetric unit showed there was slight differences in positions due to crystal contacts. In RmlC, the β-phosphate was positioned in the same way as the β-phosphate in the DdahB GDP complex. The location of the hexose ring in RmlC is very different from that seen for the ordered mannose in the DdahB complex. In RmlC and cupins, in general, the active site is located in a cavity in the center of the structure.

The experimental location of the mannose ring in the DdahB structure was difficult to reconcile with catalysis. We therefore relied on RmlC to guide the identification of the active site in both DdahB and MlghB. Based on *St. suis* and *S. enterica* RmlC, we identified the following residues as forming the active site of MlghB and DdahB: His 67, Lys 74, Asn 121, Tyr 132, Tyr 134, and Asp 173 (Fig. 3, *B* and *C* and Table S1). These residues are all conserved in RmlC where they are numbered His 76, Lys 82, Asn 127, Tyr 138, Tyr 140, and Asp 180. Most are also structurally conserved in *S. enterica* RmlC, and their expected functions are summarized in Table S1.

Although the structures of DdahB and MlghB are almost identical, there is a very clear difference at the C-terminus. After Leu 169, the main chain of MlghB forms a helical turn similar to RmlC that encloses the active site. In DdahB, the chain adopts a very different route and instead makes interactions across the dimer interface (Fig. 3*B*). As a result, Asp 173 which inserts into the catalytic site in MlghB is remote from it in DdahB. Notably, Glu 171 of DdahB makes a hydrogen bond to the backbone amide of Leu 27∗ (from the other subunit). This interaction would not be possible in MlghB, which has the shorter Asp side chain at 171.

### Site-directed mutagenesis of DdahB and MlghB

**Figure 4:**
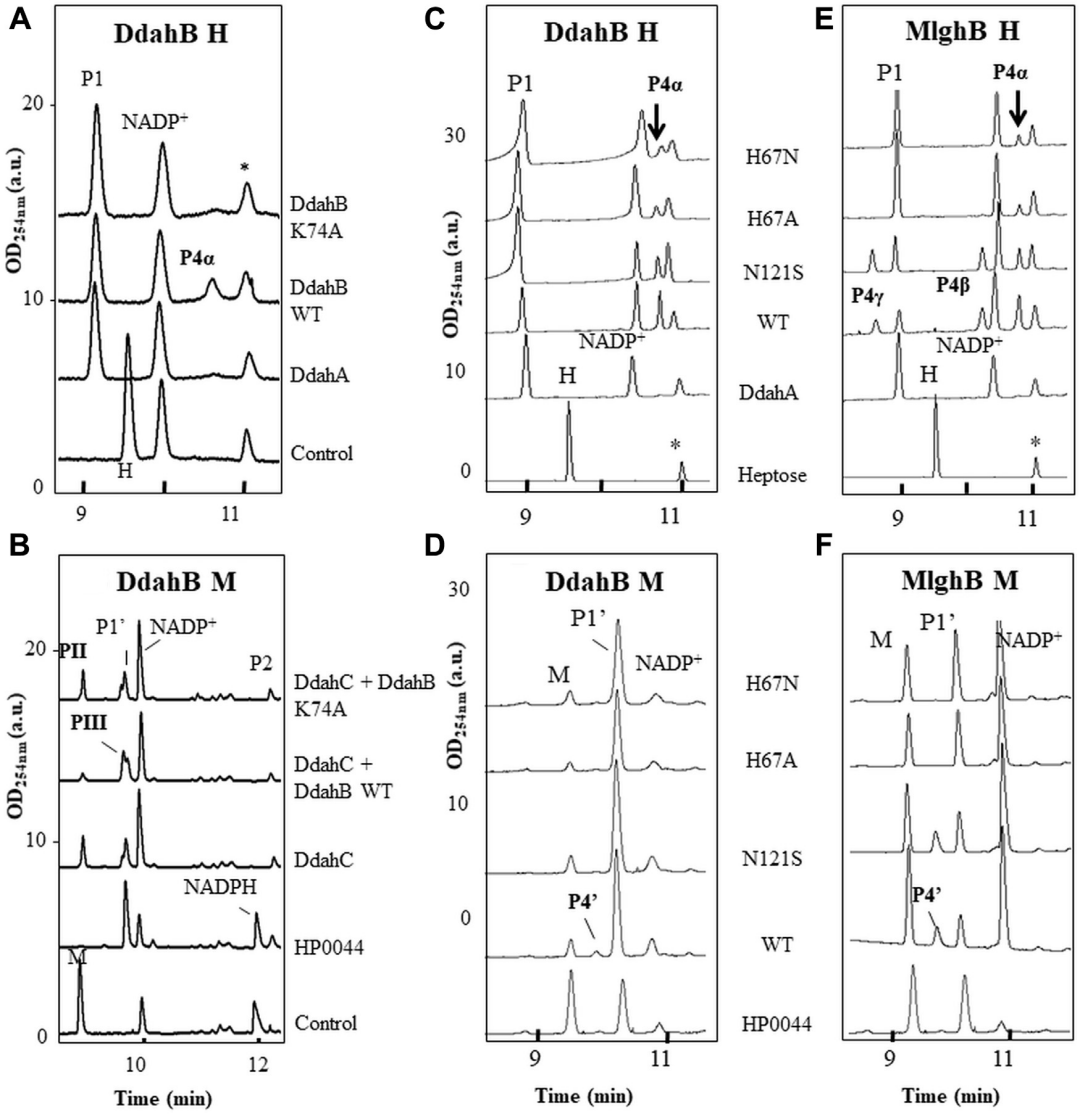
**Catalytic activity of K74A, H67N/A, and N121S DdahB and/or MlghB.***A*, reactions containing 23.2-μM DdahA, 0.2-mM GDP-*manno*-heptose (H), 0.2-mM NADP^+^, and 0.4-μM DdahB were incubated for 45 min. ∗ denotes an impurity present in the heptose preparation and that serves as an internal standard. The reaction product P4α is observed for WT DdahB but not for K74A mutated DdahB. *B*, reactions containing 5-μM HP0044, 1-mM GDP-mannose (M), and 1-mM NADPH/^+^ (50/50 %/%) were incubated for 2 h to convert all mannose in P1’ before incubation with 4-μM DdahB and 1 μM of DdahC for 5 h. DdahC was added to stabilize any P4’ generated by DdahB. Peaks PII and PIII correspond to DdahC reduction products, stemming from P1’ and P4’, respectively, as demonstrated later in the study. Formation of PIII is observed in reactions comprising WT DdahB, but not K74A DdahB, indicating impaired P4’ formation by K74A DdahB. *C* and *E*, reactions containing 0.17-mM heptose, 1.5-μM DdahA, 0.13-mM NADP^+^, and 0.1-μM epimerase were incubated for 30 min. The effect of the mutations is assessed by comparing the amount of epimerization product formation (P4α, P4β, P4γ) between mutated and WT enzymes. *D* and *F*, a 50-μl reaction containing 0.77-mM mannose and 0.2-μM HP0044 was incubated for 90 min to generate P1’ (∼50% conversion obtained). Aliquots of 8 μl were supplemented with 0.5-μM (final concentration) of DdahB and 0.1-mM NADP^+^, 0.1 μM of MlghB and 0.5-mM NADP^+^, or none and incubated for 5 h. The higher concentration of DdahB used accounts for its low catalytic efficacy on mannose. The effect of the mutations is assessed by comparing the amount of epimerization product formation (P4’) between mutated and WT enzymes. All reactions were set up in duplicates. Figure 4 shows a representative example of each reaction. Quantitation and kinetics are shown in Fig. S2.

**Figure 5:**
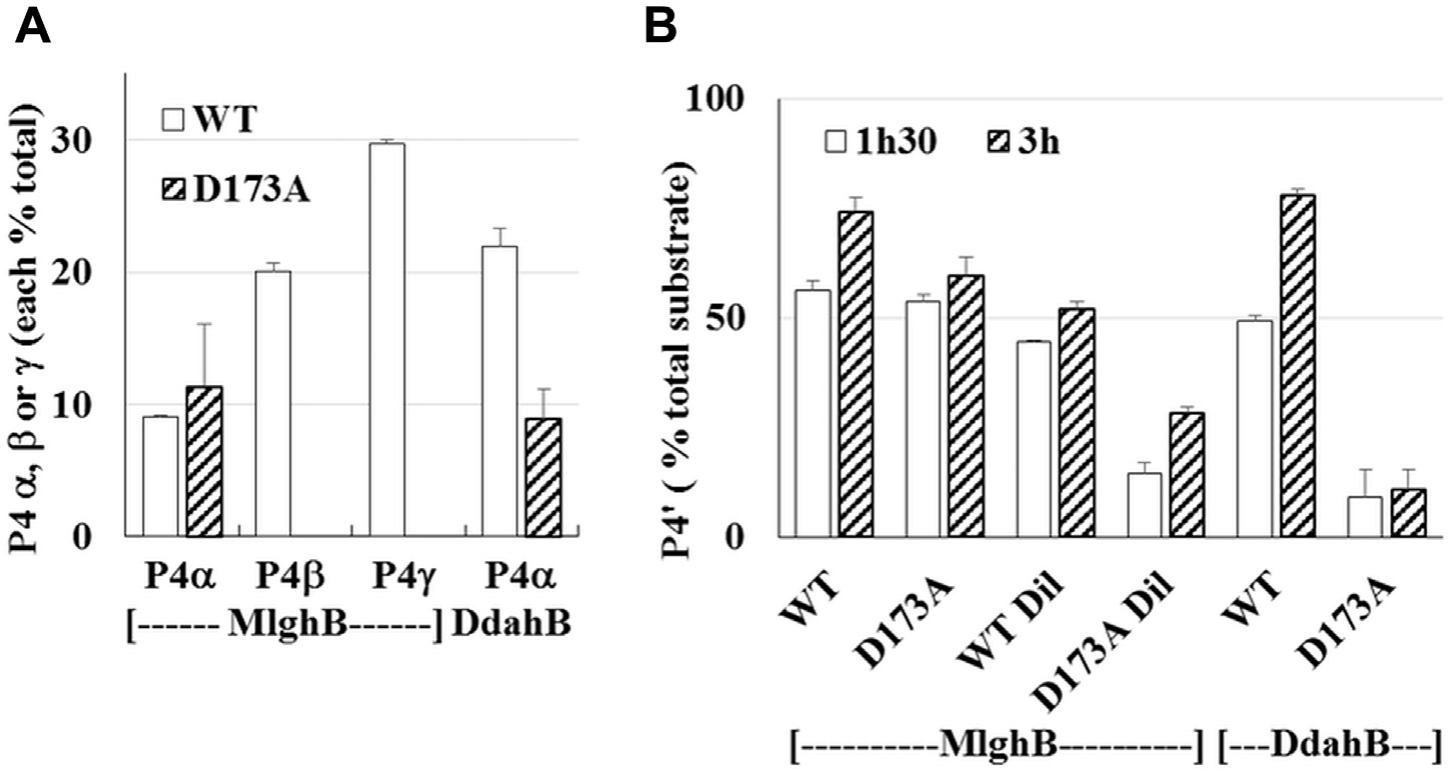
**Effect of D173A mutation on activity of DdahB and MlghB.***A*, quantitation of various epimers produced in 45 min in 10-μl reactions containing 0.25-mM heptose, 0.75-mM NADP^+^/H mix, 7.5 μM of DdahA, and 0.40 μM of epimerase. Additional data showing conversion as a function of time and enzyme concentration are shown in Fig. S3. Reduced amounts of P4α indicate that D173A limits C3 epimerization activity of DdahB. Lack of P4β and P4γ indicates that D173A abrogates C5 epimerization of MlghB. *B*, mannose conversion in reactions containing 0.1-mM mannose, 0.34-mM NADP^+^/H mix, 0.27-μM of HP0044, and 0.65-μM DdahB, 0.4-μM MlghB, or 0.08-μM MlghB (Dil sample) and that were incubated for 1 h 30 min and 3 h. DdahB was used at a higher concentration than MlghB to make up for its poor activity on mannose. Reduced amounts of P4’ indicate that C3 epimerization of mannose is affected in both mutated enzymes. In all panels, each bar represents the average of 2 determinations using 2 different batches of overexpressed enzymes.

We assayed mutants of residues His 67, Lys 74, Asn 121, Tyr 132, Tyr 134, and Asp 173 in DdahB and MglhB. All data are summarized in Table 2. Consistent with RmlC data, mutation of Lys 74 abolished all activity in DdahB for both substrates (Fig. 4, *A* and *B*). Mutations in the predicted His 67/Asp 173 dyad showed unexpected effects. Mutations H67A and H67N only reduced C3 epimerization of the heptose P1 to 30 and 25% of WT, respectively, in both MlghB and DdahB as revealed by lower amounts of P4α C3 epimer and eliminated MlghB’s C5 heptose epimerization (no P4β or P4γ), thus showing a site-specific effect (Fig. 4, *C* and *E* and Fig. S3). The retention of C3 epimerization activity on heptose after mutation of His 67 is in contrast to RmlC studies, which identified this residue as essential (16, 17). However, the essential nature of this residue appears substrate dependent because no activity was detected on mannose-based P1’ substrate (no P4’, Fig. 4, *C* and *D* and Fig. S3). Mutation D173A did not abolish C3 epimerization of P1 for either enzymes because product P4α was observed but reduced it to 40% of WT level in DdahB (Fig. 5*A*). MlghB D173A produced as much P4α as WT, but its C5 epimerization was abolished (no P4β or P4γ). This indicates that C3 and C5 epimerization of heptose by MlghB can be decoupled by simple mutation of D173. Overall, the total substrate conversion only amounted to 17% of WT levels in MlghB D173A. For the P1’ mannose substrate, C3 epimerization was reduced as indicated by decreased P4’ product formation, more so in DdahB than in MlghB, also showing differential effects of the mutation depending on the substrate (Fig. 5*B*). The reduced catalytic performance is consistent with RmlC data, which suggest a role of the Asp as a basicity enhancer for the catalytic His residue as opposed to being a direct catalytic residue. The stronger effects on DdahB may relate to the different position of this residue with regard to the catalytic histidine.

**Table 2:** Summary of SDM data obtained for the epimerases DdahB and MlghB both on heptose and mannose substrates

Mutated AA	Role in RmlC (15, 16, 17)	Effect on epimerization activity at C3 and C5 for				
		RmlC M	DdahB H	MlghB H	DdahB M	MlghB M
K74A	Enolate stabilizer	C3 X C5 X	C3 X	Not tested	C3 X	Not tested
H67A	Essential catalytic base for C3, C5 epimerization	C3 X C5 X	C3 ↓↓	C3 ↓↓ C5 X	C3 X	C3 X
H67N	Essential catalytic base for C3, C5 epimerization	C3 X C5 X	C3 ↓↓	C3 ↓↓ C5 X	C3 X	C3 X
D173A	Part of H67/D173 dyad, base enhancer	C3 ↓↓↓↓ C5 ↓↓↓↓	C3 ↓↓	C3 ↓ C5 X	C3 ↓↓↓	C3 =, ↓
N121S	Substrate binding at O4	Not tested	C3 =, ↓	C3 ↓ C5 ↑	C3 X	C3 =
Y134F	Essential catalytic base for C3, C5 epimerization	C3 X C5 X	C3 ↓↓↓	C3 X C5 X	C3 ↓↓	C3 ↓↓↓↓
Y132F	Unknown	Not tested	C3 ↓	C3 ↓↓↓ C5 ↓↓	C3 ↓↓	C3 ↓↓
YY/FF	Unknown	Not tested	C3 ↓↓↓	C3 X C5 X	C3 ↓↓	C3 X

AA, amino acid; H, heptose; M, mannose; X, inactivated enzyme.

↓ to ↓↓↓↓: modest to severe decreased enzymatic activity; =: no change in enzymatic activity; =, ↓: moderate activity decrease noted only under low enzyme concentrations and/or short incubation times.

**Figure 6:**
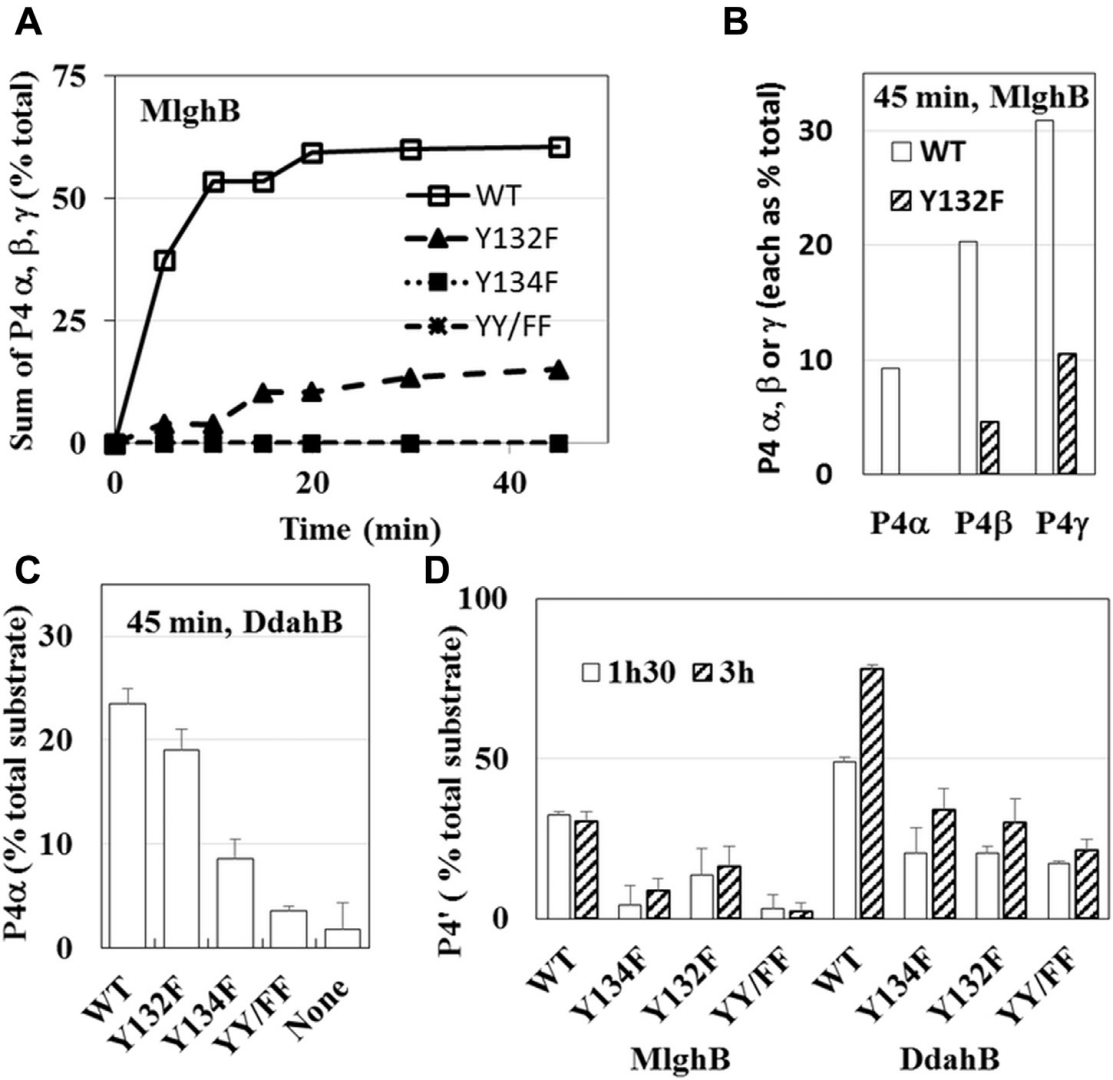
**Effect of Y132F, Y134F, and double mutation (YY/FF) on catalysis by the epimerases.** The reaction conditions are as described for Figure 5. *A* and *B*, effects on MlghB showing the sum of all heptose-derived products as a time course (*A*) and the proportions of various products made in WT and Y132F mutant at 45 min (*B*). *C*, effects on DdahB shown at 45 min for heptose conversion into the single epimerization product P4α. *D*, effects on both enzymes on mannose catalysis at 1 h 30 mins and 3 h as assessed by variations in their unique reaction product P4’. DdahB and its mutants were more concentrated than MlghB and its mutants to compensate for DdahB’s poor activity on mannose. In panels *C* and *D*, each bar represents the average from 2 batches of enzymes. Additional supporting data are shown in Figs. S4 and S5.

The study of conserved predicted catalytic Tyr 134 and nearby Tyr 132 showed enzyme-specific effects. MlghB Y134F was totally inactive on heptose (no P4α, P4β, or P4γ in Fig. 6*A* and Fig. S5) and showed reduced activity on mannose (10% of WT level of P4’, Fig. 6*D*). These results are similar to those obtained for Tyr 140 in RmlC (16), supporting a role as proton donor, but were in contrast to the DdahB Y134F mutant where conversion catalysis was retained and only reduced for both heptose-based P1 (5–40% of WT P4α level) and mannose-based P1’ (50% of WT P4’ level). We surmise that Tyr 132 may also participate in catalysis, either along with or instead of Tyr 134 and may determine substrate specificity. Accordingly, MlghB Y132F showed a reduction in heptose-based P1 conversion (25% of WT level of P4α, P4β, and P4γ after 45 min, Fig. 6*A* and Fig. S5) although epimerizations at C3 and C5 positions were observed (Fig. 6*B*, presence of C5 epimer P4β and double epimer P4γ). MlghB Y132F also retained around 50% of WT C3 epimerization of the mannose-based P1’ substrate shown by P4’ formation (Fig. 6*D*). The stronger effect of the Y132F mutation on heptose *versus* mannose catalysis indicates that Y132 is a determinant of MlghB’s substrate specificity, probably ensuring proper heptose positioning *via* interactions with its hydroxyl side chain. A role for interaction with C6 and O6 of the substrate has been proposed for the equivalent residue in *St. suis* RmlC (16), and the presence of the extra bulky C7 substituent in heptose is consistent with a different level of interaction with the two substrates. In contrast, DdahB Y132F retained between 50 and 90% of WT activity on the heptose-based P1 substrate (forming P4α) and 45% of WT on the mannose-based P1’ substrate (forming P4’) (Fig. 6, *C* and *D*). The double DdahB mutant Y132F/Y134F was active on both substrates (forming P4α and P4’), showing performances similar to that of the single Y134F mutant and suggesting that the activity of the Y134F DdahB was not due to compensation by Tyr 132.

Finally, Asn 121 which is equivalent to Asn 127 of *St. suis* RmlC and to His 120 in *S. enterica* RmlC was studied for its predicted role in binding the O4 of the 4-keto substrate and in favoring proton abstraction from C3 and C5 (16) (Table S1). Considering that the O4 position may be altered with mannose *versus* heptose-based substrate, Asn 121 may contribute to substrate specificity. In addition, in *S. enterica* RmlC, His 120 may also form an extra catalytic dyad with Asp 84 (15, 16, 17), which would correspond to Asn 121/Gln 85 in MlghB and DdahB. This is supported by the structure that shows that their side chains point toward one another. N121S mutants of both proteins retained their catalytic activity on heptose, which suggested that although this residue may play a role in positioning the substrate, it is not directly involved in catalysis (Fig. 4, *C* and *E* and Fig. S3) and thus N121 is not forming the predicted N121/Q85 catalytic dyad. MlghB N121S showed less accumulation of C3 epimer P4α, suggesting either slightly decreased efficacy of C3 epimerization or enhanced C5 epimerization that prevents P4α accumulation (Fig. 4*E* and Fig. S3). In contrast, the N121S mutation had very limited effect on P4’ formation from mannose by MlghB (Fig. 4*F* and Fig. S3) while it abrogated DdahB’s already limited activity (Fig. 4*D*). This suggests that N121 influences substrate specificity differentially in both enzymes and that the N121S mutations reinforces DdahB’s specificity for heptose.

### Enzymatic activity of MlghC and DdahC

**Figure 7:**
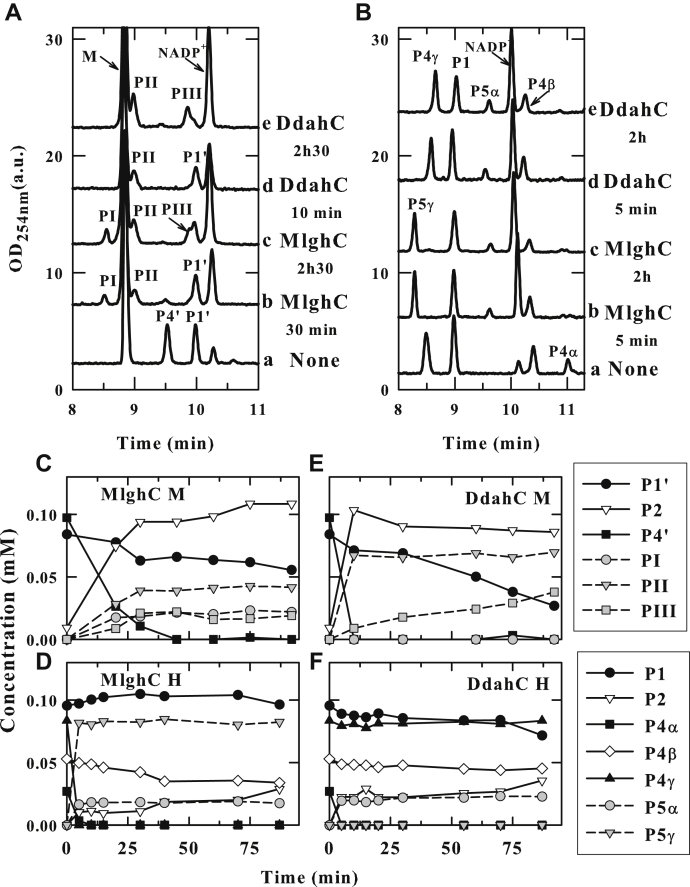
**Specificity of MlghC and DdahC for heptose *versus* mannose.** DdahA and MlghB were incubated with GDP-*manno*-heptose or GDP-mannose; both enzymes were removed by ultrafiltration, and the products were incubated with MlghC or DdahC in the presence of NADPH. All reactions comprise residual DdahA and MlghB reaction products as defined in Figures 1 and 2. *A* and *B*, CE traces of reactions performed on GDP-6-deoxy-4-keto-mannulose (*A*) or heptulose (*B*). PI, PII, and PIII denote new products arising from mannose-based P4’ and P1’. P5γ and P5α are heptose-based reduction products of MlghC and DdahC, respectively, as described in Figure 1. *C*–*F*, time course of substrate conversion for reactions performed in parallel with heptose (*D* and *F*) *versus* mannose (*C* and *E*) for MlghC (*C* and *D*) or DdahC (*E* and *F*). The procedure and stoechiometries used to achieve equimolar substrate amounts for all reactions are detailed in Experimental procedures section. This is a representative example of 2 independent experiments. CE, capillary electrophoresis.

To assess the specificity of MlghC and DdahC, substrates P4α, P4β, and P4γ were generated from GDP-*manno*-heptose incubated with DdahA and MlghB, while P4’ was generated similarly but starting from GDP-mannose. These substrates along with residual starting GDP-mannose or GDP-*manno*-heptose and their corresponding DdahA products (GDP-4-keto, 6-deoxy-mannose P1’, or *manno*-heptose P1) were isolated using ultrafiltration and incubated in equimolar amounts with MlghC or DdahC in the presence of NADPH cofactor (Fig. 7).

As established previously, the heptose-derived substrates were fully catalyzed within 5 min and without degradation, P4α to P5α by DdahC, and P4γ to P5γ by MlghC, respectively (Fig. 7, *B*, *D* and *F*). In MlghC reactions, only the double epimer P4γ is reduced but the amounts of products P4α and P4β also decrease because of their conversion to P4γ by residual MlghB (Fig. 1). Both DdahC and MlghC also catalyzed the reduction of the mannose-based substrate P4’, but instead of one product, MlghC gave rise to three products, denoted PI, PII, and PIII, while DdahC gave rise to two of these products PII and PIII (Fig. 7, *A*, *C* and *E*). P4’ was completely consumed after 45 and 10 min for MlghC and DdahC, respectively. However, a high level of degradation of the P4’ substrate into GDP (P2) was observed, indicating that reduction of P4’ was slow. Thus, both MlghC and DdahC show a strong preference for the C7 substrate over a mannose-based substrate.

**Figure 8:**
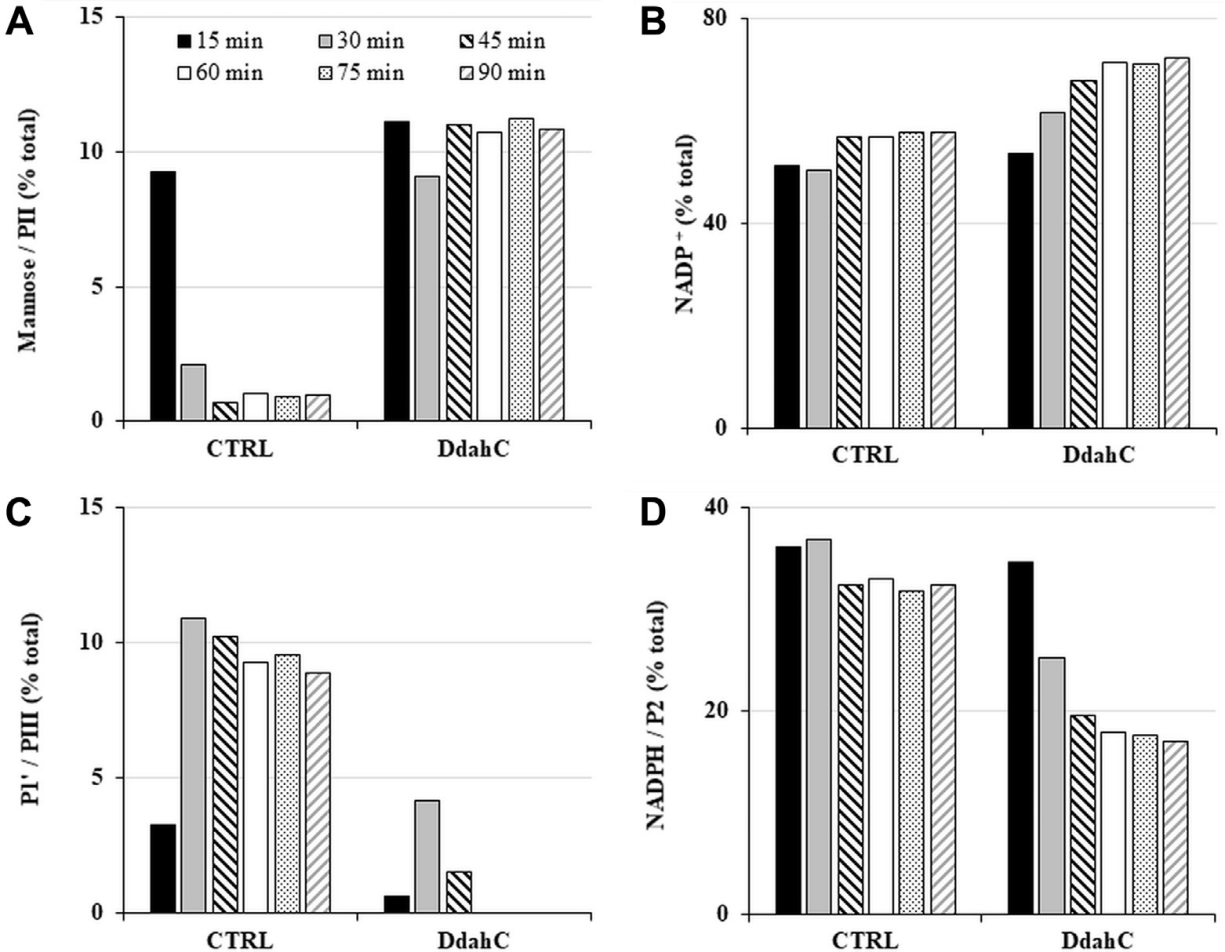
**Kinetic analysis highlighting the activity of the DdahC reductase on the dehydrated product of GDP-mannose P1’ to make product PII in the absence of epimerase.** Reactions comprising 0.1-mM GDP-mannose, 0.27-μM dehydratase HP0044, 2.6-μM DdahC, and 7.5-mM NADPH/^+^ mix were incubated at 37 °C from 15 to 90 min. The samples were run on CE, and the area under each peak integrated to calculate the relative proportions of all components present. Because PII overlaps with mannose and PIII overlaps with P1’, their potential formation was assessed in panels *A* and *C* by comparing the kinetics of product formation between reactions comprising DdahC and control reactions (CTRL) devoid of DdahC. When control reactions devoid of DdahC show complete disappearance of mannose in the mannose/PII CE migration area, then the signal observed in the mannose/PII area of reactions comprising DdahC can be interpreted as the formation of product PII. Thus, DdahC made PII out of P1’ in epimerase-free reactions analyzed in this figure, and by extension, it had made PIII out of P4’ in reactions comprising an epimerase (that generates P4’, Fig. 7*E*). The concomitant formation of NADP^–^ and disappearance of NADPH were also recorded (panels *B* and *D*) to ascertain the occurrence of the reduction reaction in the presence of DdahC. This figure is a representative example of 2 independent experiments optimized for the sake of identifying the substrates used and the products generated. CE, capillary electrophoresis.

**Figure 9:**
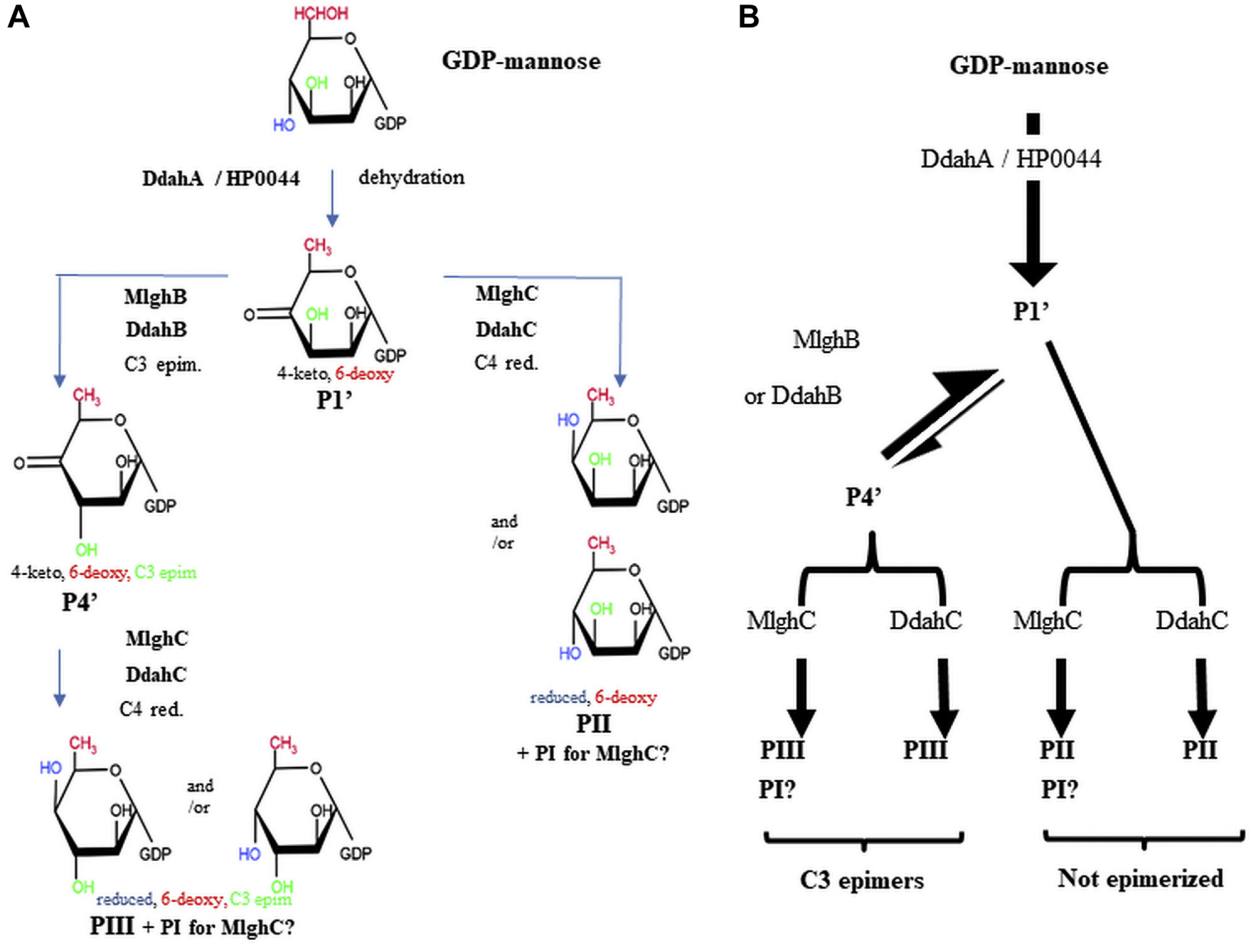
***In vitro* GDP-mannose modification pathways.***A*, pathways showing the various configurations of sugars formed. *B*, simplified pathways. GDP-4-keto, 6-deoxy-mannulose P1’ is produced from GDP-mannose using dehydratases DdahA (low yield) or HP0044 (high yield). Reaction of P1’ with MlghB or DdahB yields the same single product P4’, which we surmised, corresponds to the C3 epimer. This epimer can be reduced by MlghC or DdahC to form PIII. The position of the C4 OH group on PIII is unknown. MlghC and DdahC can also reduce the original P1’ into PII, for which the position of the C4 OH group is also not known. In addition, MlghC generates small amounts of PI from reactions comprising both P1’ and P4’. Its precise origin (P1’ *versus* P4’) and epimerization status have not been determined. PI corresponds to the complementary C4 epimer of PII or of PIII.

Because the stereospecific reduction of P4’ should yield only one product but up to three products (PI, PII, and PIII) appeared, we reasoned that the reductases may also be reducing other molecules in the reaction mixture, including the nonepimerized residual product P1’. To test this, DdahC was incubated directly with P1’ (generated from GDP-mannose *in situ* by incubation with GDP-mannose C4, C6 dehydratase HP0044) in the absence of epimerase. The reaction was assessed by comparing the kinetics of change in the amounts of the various reaction components between reactions comprising DdahC and control reactions devoid of DdahC. This comparison was required because GDP-mannose itself overlaps with PII, and P1’ overlaps with PIII, precluding simple analysis. In the absence of DdahC, all GDP-mannose was converted into P1’ after 45 min (Fig. 8). We therefore assumed that there was no GDP-mannose remaining underneath the PII peak after 45 min; thus, any peak at PII is entirely due to PII formation. Consumption of NADPH and increase in NADP^+^ were observed, indicating a reduction reaction. No PIII or PI was detected even after prolonged incubation under these conditions. We conclude DdahC has reduced the nonepimerized P1’ substrate into PII (Fig. 9). This implies that product PIII has arisen from conversion of P4’ by DdahC (Fig. 9). We noted PIII had continued to appear even after P4’ was consumed and surmised that residual MlghB present in the reaction continued to create fresh P4’ by epimerization of P1’ which is also present. The presence of residual MlghB despite ultrafiltration was also noted in parallel GDP-*manno*-heptose reactions where low levels of MlghB-mediated interconversion of P4α, P4β, and P4γ were observed when a reductase was added to consume one of the epimers (Fig. 7*D*).

Because MlghC produced PII and PIII when incubated with a mixture of P1’ and P4’ substrates (Fig. 7*C*), we concluded that MlghC reduces P1’ into PII and P4’ into PIII (Fig. 9). MlghC also produced some PI, but we have not been able to conclusively identify the nature of PI, beyond confirming that it is a reduced GDP-hexose based on its mass, nor have we established whether it results from P1’ or P4’.

### Structural studies of MlghC and DdahC

**Figure 10:**
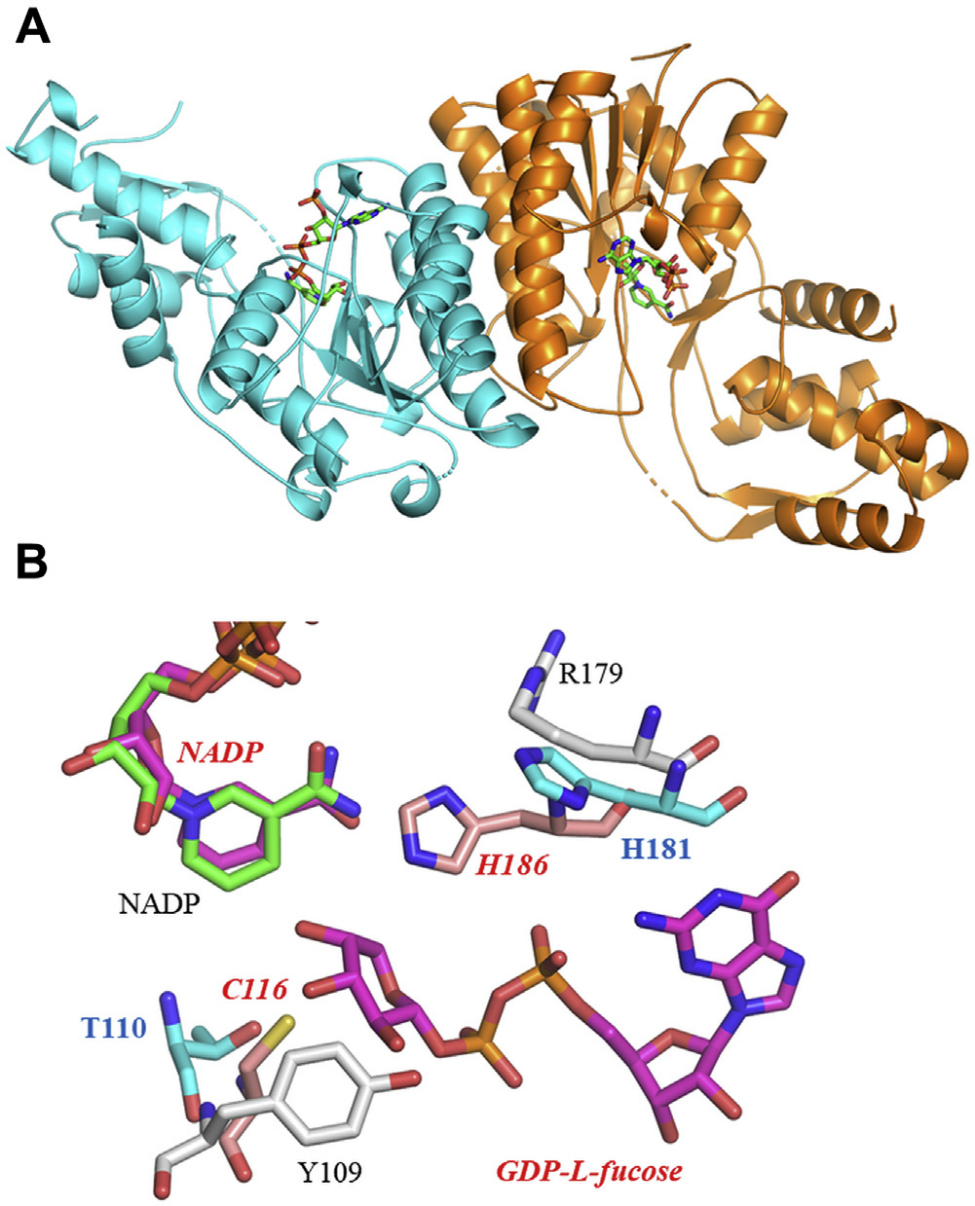
**Structural characterization of the reductases DdahC and MlghC.***A*, the dimer of DdahC shown as a *cartoon*, with one monomer in *orange* and the other in *cyan*. NADP is shown as *sticks* with carbons *green*, oxygen *red*, nitrogen *blue*, and phosphorous *orange*. *B*, the active site of MlghC and DdahC, with NADP colored as in panel *A*. Amino acid side chains are labeled and shown with carbon atoms of MlghC colored *gray* (Y/R dyad) and of DdahC in *blue* (T/H dyad). The C/H catalytic dyad of GFS (RCSB 1BWS) is shown with side chains in *salmon* and labeled in *red*. GDP-L-fucose from the GFS structure is also shown (labeled in *red*). GFS, GDP-L-fucose synthase.

A crystal of MlghC diffracted to 1.66-Å resolution and belonged to space group *P*1 2_1_ 1, with two chains in the asymmetric unit. A crystal of DdahC diffracted to 2.08 Å resolution, in space group *P*4_2_ 2_1_ 2, with one chain in the asymmetric unit. In the MlghC structure, residues 1 to 263, 268 to 304, and 310 to 344 in chain A and amino acids 0 (from tag) to 172, 179 to 202, 220 to 263, 268 to 305, and 310 to 344 in chain B were placed in density; an additional N-terminal residue resulting from the expression construct was located, and the C-terminus 345 to 347 was disordered. The final DdahC structure comprised residues 1 to 265, 268 to 308, and 313 to 352; the C-terminal residue 353 was disordered (Fig. 10*A* and Table 3).

**Table 3:** Additional crystallographic parameters for DdahC and MlghC

Parameter measured	DdahC	MlghC NADP
Data collection		
Space group	P4_2_2_1_2_1_	P2_1_
Wavelength	1.54	0.982
Unit cell dimensions		
a, b, c (Å)	131.8, 131.8, 50.2	57.8, 132.0, 59.3
α, β, γ (°)	90, 90, 90	90, 105.8, 90
Resolution (Å)^a^	99–2.08 (2.14–2.08)	66.0–1.66 (1.70–1.66)
R_merge_^a^	0.163 (0.61)	0.059 (0.76)
I/σI^a^	11.5 (3.3)	11.8 (1.2)
CC_1/2_^a^	0.9 (0.8)	1.0 (0.5)
Completeness^a^	100 (100)	99.2 (99.1)
Redundancy^a^	12.5 (7.9)	3.7 (3.1)
Refinement		
Resolution (Å)^a^	33–2.08 (2.14–2.08)	66–1.66 (1.70–1.66)
Reflections^a^	25,782 (1861)	94,907 (7016)
R_work_/R_free_ %^a^	19.0/23.2 (24.1/29.7)	17.3/20.3 (32.4/33.1)
No. of atoms		
Protein	2810	5188
Cofactor	-	96
Water	87	412
Residual B factors (Å^2^)		
Protein	26	25
Ions/buffer	-	-
Water	26	36
Ligand	-	29
RMSD		
Bond lengths (Å)	0.019	0.014
Bond angles (°)	1.9	1.5
Ramachandran		
Favored (%)	98	98
Outliers (%)	0	0
	7ANH	7ANC

a Values in parenthesis are for the highest shell.

The structures of MlghC and DdahC are, given the nearly 60% sequence identity, highly similar (RMSD 1.3 Å over 333 residues) (Fig. S7 and Table S5). The monomer (MlghC numbering) is formed from two domains: the large domain (residues 1–171, 236–280, 310–329) and a smaller C-terminal domain (172–235, 281–309, 330 to C-terminus). The large domain contains the characteristic Rossman fold and is composed of five β-strands, six α-helices, and two helical turns. The smaller domain is composed of three β-strands and three α-helices. In the DdahC crystal, there is no electron density for the NADP+ cofactor. In MlghC, electron density shows that each monomer has a bound NADP+ (clearly not NAD+) cofactor consistent with previous biochemical analysis (3, 4). The cofactor is located within the Rossman fold and anchored by a series of contacts with the protein.

Although DdahC has crystallized as a monomer within the asymmetric unit, SEC-MALS of both proteins indicated that both are dimers (Fig. S1, *C* and *D* and Table S3). Analysis of the DdahC and MlghC structures with the PDBePISA server suggests that both proteins are found as dimers in the crystal. The MlghC dimer is that found in the asymmetric unit with monomers related by a noncrystallographic twofold axis while the DdahC dimer is generated by twofold rotation that arises from crystal symmetry. Superposition of these dimers confirms that both have the same arrangement. The dimer interface comprises three α-helices in the large domain and the connecting loops. Two helices from each monomer form a four helical bundle in the center of the dimer.

Both enzymes belong to the short chain dehydrogenase reductase (SDR) class of enzymes, typified by the UDP-galactose/glucose epimerase. Typically, SDR enzymes have a constellation of three catalytic residues Ser, Tyr, and Lys, although the conservation is not absolute. In MlghC and DdahC, these are found as Ser 107, Phe 136, Lys 140 and Ser 108, Tyr 137, Lys 141, respectively (Table S2). The substitution of Tyr to Phe is unusual, but there are other examples of SDR enzymes which lack a Tyr (26, 27). Superposition of the two enzymes reveals some other differences at the active site; notably, MlghC has Tyr 109, which points into the active site, whereas DdahC has Thr 110 at the equivalent position and its hydroxyl group points away from the active site (Fig. 10*B*).

Searching for structural homologues with SSM reveals over 150 structural matches, reflecting the common nature of this fold with sequence identities ranging from 10 to 30%. The closest structural homologue to both enzymes is the GMER from *E. coli* (PDB 1e6u, 315 residues RMSD MlghC 1.9/DdahC 2 Å). This enzyme has the Ser 107, Tyr 136, and Lys 140 triad at the active site and has the same dimeric arrangement, but as yet no substrate or product complex has been reported for this enzyme. The human enzyme known as GFS (a paralogue of *E. coli* GMER) has the same fold as MlghC (Fig. S8). MlghC and DdahC are also structurally related to GDP-mannose 4,6 dehydratase (RCSB 1db3) and to the dDTP-4-keto-L-rhamnose reductase RmlD, the final enzyme in dTDP-L-rhamnose pathway (28). Equivalent residues in GFS, RmlD, MlghC, and DdahC are indicated in Table S2. All RMSD values are summarized in Table S5.

These enzymes all share the same SDR fold and adopt the same dimeric structure, with the exception of RmlD, which has a unique Mg^2+^-mediated dimerization arrangement. Where the co-factor is present, it makes identical interactions with the protein in all the structures. The GFS and RmlD co-complex structures allow us to locate the likely binding site in MlghC and DdahC. The guanidine binding site in GFS is formed by Val 187 and Trp 208. In MlghC Val 180 and Trp 231 (DdahC Val 182, Trp 232) occupy the same structural location, suggesting they would play the same role. MlghC has an open pocket lined by Pro 67-Cys 68, Asn 165, and Arg 179 (DdahC Ala 67-Gly 68, Asn 166, His 180). Compared with both GFS and RmlD, the pocket in MlghC (and DdahC) is more open, presumably reflecting the large C7 substrate. We suggest this larger pocket has the flexibility that allows the correct positioning of the C4 keto in both the C6 and C7 substrates.

### Site-directed mutagenesis and analysis of reductases

**Figure 11:**
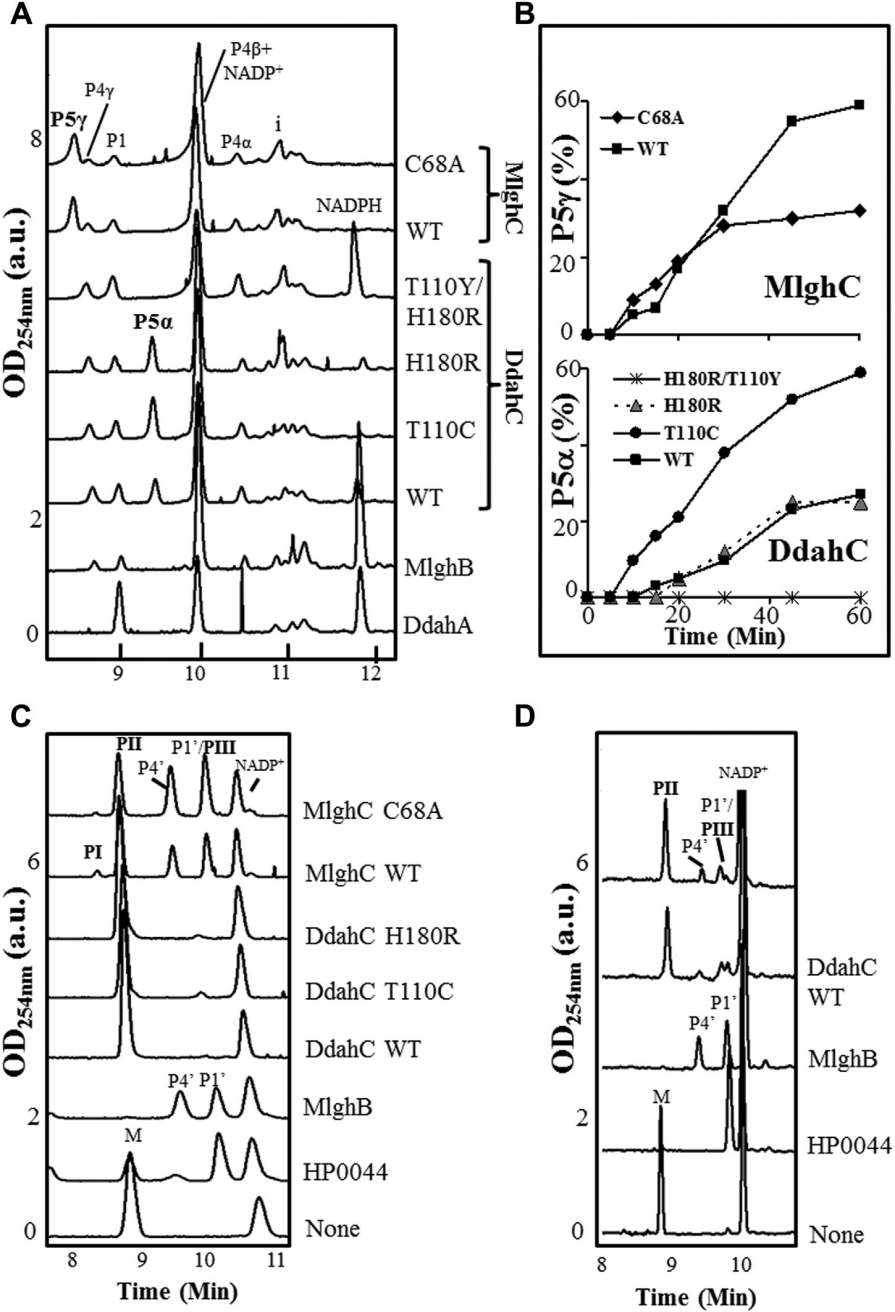
**Catalytic activity of mutated reductases.***A* and *B*, CE profiles at 30 min and kinetics for reductase activity on heptose whereby the substrates P4α, P4β, and P4γ were generated by DdahA and MlghB. A master reaction containing 0.37 mM of heptose, 0.5 mM of NADPH/^+^, and 0.4 μM MlghB was incubated for 30 min. For panel *A*, 0.1 μM (final concentration) of reductase was added to 9 μl of master mix and the volume brought to 10 μl before further incubation for 30 min. For panel *B*, the master mix was ultrafiltered to remove MlghB and used to perform the reductase kinetics on fixed amounts of P4α, P4β, and P4γ substrates incubated for up to 60 min with 0.5 μM (final concentration) of reductase. Reductase activity is denoted by formation of peaks P5α for DdahC or P5γ for MlghC from epimers P4α and P4γ, respectively. The data shown are from one experiment and are representative of independent repeats performed with different enzyme batches and showing similar trends. *C* and *D*, activity on GDP-mannose whereby the substrate P4’ was generated by HP0044 and MlghB. For panel *C*, reactions contained 0.3 mM of mannose, 0.3 mM of NADPH/^+^, 0.2 μM of HP0044, 0.4 μM of MlghB, and 0.5 μM of reductase in 10 μl and were incubated for 5 h. The data shown are representative of 2 independent repeats. For panel *D*, reactions contained 0.1 mM of mannose, 0.27 μM of HP0044, 0.34 mM of NADPH/^+^, 0.65 μM of epimerase MlghB, and 1 μM of reductase in 10 μl and were incubated for 1 h 45 mins. The different stoichiometry in panel *D* aimed at slowing down the formation of the epimer substrate to maximize its reduction by more abundant DdahC. The reactions were set in duplicates. and one representative trace is shown for each in panel *D*. For both panels *C* and *D*, complete usage of mannose substrate in the control reaction (MlghB trace, no reductase) ensures that the peak in the overlapping mannose/PII area of reductase-containing reactions is the reduction product PII.

We selected C68 of MlghC for site-directed mutagenesis in advance of our structural work using GFS as a guide for structural modeling. The structure of MlghC in fact showed that C68 is buried and remote from the NADP^+^ binding site, and C68A showed no effect on product or substrate specificity (across heptose epimers and for mannose *versus* heptose) (Fig. 11, *A* and *C*). The C68A mutation only led to a lower yield of product formation at long incubation times (>30 min), which may reflect decreased enzyme stability (Fig. 11*B*). We mutated the T110/H180 dyad in DdahC with a T110C mutant, so it had a C/H GFS-like dyad, a double T110Y/H180R DdahC mutant to have a Y/R MlghC-like dyad and an H180R mutant with a mixed T/R dyad (Table S2, Fig. 10*B*). We failed to obtain a T110Y mutant designed to generate a mixed Y/H dyad. All data are summarized in Table 4. Both the T110C and H180R DdahC mutants catalyzed the reduction of P4α to P5α but did not reduce the P4β or P4γ also present in the reaction, thus preserving substrate specificity. The T110C mutant (Fig. 11, *A* and *B*) had enhanced efficiency, while H180R was indistinguishable from WT. The same results were obtained when reactions were performed in the presence of the P4α epimer only (generated using DdahB) (Fig. S9).

**Table 4:** Summary of activity data obtained on WT and/or mutated reductases DdahC and MlghC on heptose- and mannose-based substrates

Enzyme	Status	Final AA	Heptose	Man. P4’	Man. P1’	Expectation
GFS	WT	H179 C109	n/a	n/a	Normal substrate	Catalytic dyad serving as general acid and base for epimerization at C3 and C5.
MlghC	WT	R180 Y108	P4γ to P5γ	PI + PIII	PII	Catalytic dyad
DdahC	WT	H180 T110	P4α to P5α	PIII	PII	Catalytic dyad
DdahC	T110C	H180 C110	P4α to P5α ↑↑	PIII =	PII =	GFS-like dyad. Enhanced mannose usage, especially nonepimerized P1’.
DdahC	H180R	R180 T110	P4α to P5α =	PIII =	PII ↑↑↑	Intermediate toward MlghC dyad. Switch of heptose epimer specificity to P4γ and decreased mannose usage.
DdahC	H180R T110Y	R180 Y110	Inactive	PIII =	PII =	MlghC-like dyad. Switch of heptose epimer specificity to P4γ and decreased mannose usage.

↓, modest decrease in enzymatic activity; =, no change in enzymatic activity; ↑ to ↑↑↑, moderate to strong increase in enzymatic activity; Heptose: heptose-based substrates; Man., mannose-based substrates; n/a, not applicable.

Final AA refers to the amino acids present at the site of interest in the WT or mutated enzyme.

Status refers to either WT or mutated enzyme.

Data under columns Heptose, Man. P4’, and Man. P1’ show observed catalytic activity, which differed from original expectations.

The experiment was repeated with P4’ that was generated *in situ* from GDP-mannose by a combination of HP0044 and MlghB. When a slight excess of DdahC was used (1.25/1 DdahC/MlghB molar ratio), neither P4’ nor P1’ was observed and only PII was seen; thus, DdahC appeared to have reduced P1’ faster than MlghB can generate P4’. This rapid consumption of P1’ was not affected by the mutations (Fig. 11*C*). When a limiting amount of DdahC was used to slow down the reduction (DdahC/MlghB molar ratio decreased to 0.5/1 while keeping MlghB constant), MlghB produced P4’ and both WT and T110C mutant DdahC generated peak PIII from P4’ (Fig. S9*B*). The T110C mutant produced twice as much PIII than WT enzyme at 1 h and hardly any PII. We conclude WT DdahC (and especially the T110 C mutant) has a strong preference for the epimerized substrate P4’ (to produce PIII) over its nonepimerized counterpart P1’ (to produce PII) (Fig. 9, Table 4).

The H180R mutant produced mostly PII (Fig. S9*B*), with some PIII appearing at 2 h only but with less efficacy than in WT DdahC. However, performance with heptose was WT like (Fig. S9*A*, Table 4), indicating proper enzyme folding and stability. This indicates that the H180R mutation did not abolish the ability to use mannose-based P4’ to make PIII but conferred preference for the nonepimerized substrate P1’, thus representing a switch in substrate specificity among mannose-based substrates.

Interestingly, the double-mutant H180R/T110Y mutant showed no activity against heptose-derived P4α (Fig. 11 and Fig. S9*A*) but retained activity against the GDP-mannose–derived substrates P1’ and P4’, with a similar catalytic power as native DdahC (Fig. 11*D*). The double-mutation H180R/T110Y thus conferred DdahC specificity for GDP-mannose–derived substrates (Table 4). The structure of DdahC shows that these mutations will compact the active site; the closure of the DdahC we propose is responsible for the altered activity.

## Discussion

Epimerization of GDP-*manno*-heptose requires the abstraction of protons at the C3 and C5 positions from one face of the sugar ring and their replacement on the other. The abstraction of an unactivated proton from a carbon atom, such as that found in *manno*-heptose, is not possible for amino acids. To achieve this, biology introduces a keto function at the C4 position; this activates the protons at C3 and C5 by lowering their pKa to within the reach of amino acids. For this reason, epimerization requires a set of distinct chemical reactions: oxidation, acid base chemistry, and reduction. In some instances, a single enzyme has evolved to carry out this entire chemistry, while for other sugar products, a series of different enzymes is required. The enzyme pairs DdahB/C and MlghB/C which result in the epimerization and reduction of the central 4-keto-*manno*-heptose are of interest as specific anti-*Campylobacter* targets. In the Ddah pathway, the initiating C4, C6 dehydratase DdahA creates the 4-keto function and is specific for the C7 sugar (2, 4). The initiating C4 oxidase for the Mlgh pathway MlghA was recently identified as Cj1427 (formerly WcaG), which requires α-ketoglutarate to support its activity on GDP-*manno*-heptose (29, 30).

The epimerase enzymes DdahB and MlghB are almost identical to each other in sequence and structure. These enzymes show a clear preference for the GDP-4-keto-*manno*-heptose (C7 sugar) over GDP-4-keto-mannose. With GDP-4-keto-*manno*-heptose, MlghB was found to be an efficient C3/C5 epimerase establishing the equilibrium of the various mono- and double-epimerized products rapidly. DdahB, while capable of producing the double-epimerized product from the C7 substrate, appeared mainly to function as a mono-epimerase of the C3 position. This difference is surprising, given the enzymes are almost identical to each other. When presented with GDP-4-keto-mannose, DdahB again epimerizes one position; by analogy with GDP-4-keto-*manno*-heptose, it is assumed to be the C3 position. With the C6 substrate, MlghB also appeared to mainly be able to produce the same mono-epimerized product as DdahB.

Although DdahB and MlghB share limited sequence homology with RmlC (∼38%), they are in fact very similar in structure and the residues identified as controlling catalysis in RmlC are found in both in DdahB and MlghB. RmlC, part of the dTDP-L-rhamnose pathway, binds a different nucleotide and operates on a C6 sugar. Although we have obtained co-complexes of DdahB and MlghB with GDP-mannose, in these complexes, the mannose portion was disordered or was located outside the presumed catalytic site. We suggest that because GDP-mannose is not the ‘authentic’ substrate, this explains the failure to give an ordered substrate complex. Using the GDP portion observed in the complexes and using superposition with the RmlC substrate complex, we were able to analyze the active sites of both MlghB and DdahB.

The first question we sought to answer was the specificity for the C7 substrate. In RmlC, the hydroxyl of Ser 65 hydrogen bonds to the O6 of the substrate, and the side chain of Phe 129 makes van der Waal contacts with C6 of the substrate. In DdahB and MlghB, Ser 65 has been replaced with Ile 56, which would provide a hydrophobic interaction for the C7 atom of GDP-4-keto-*manno*-heptose. Phe 129 has changed to His 123 in DdahB and MlghB, and the imidazole side chain could function to provide a hydrogen bond to O7 of GDP-4-keto-*manno*-heptose. These changes create a larger volume at the active site, consistent with the larger substrate. This configuration is no longer complementary with a hexose substrate, and this we propose is why the enzymes are so much less efficient with the GDP-mannose. The active site is too large, allowing the C6 substrate freedom to move around and adopt a position that is incompatible with further catalysis.

By analogy with RmlC, we predicted that His 67, activated by its hydrogen bonds to Asp 173, would function to abstract the proton from C3 on one face of the substrate. Lys 74 and Asn 121 would function to stabilize the enolate by hydrogen bonding to the O4. Tyr 134 is predicted to function as the acid, transferring a proton to the C3 position but from the opposite face, thus epimerizing the substrate. The epimerized substrate would adjust its position in the active site, allowing the protonation state to reset and the epimerization to restart at C5, once again with His 67 and Tyr 134 predicted to playing the same role. Tyr 132 also appears involved in this process in MlghB.

Site-directed mutagenesis showed that as expected Lys 74 stabilizes the enolate. Also, C5 epimerization was abrogated as expected upon mutating His 67 to Asn or Ala in MlghB. However, although C3 epimerization of GDP-4-keto-mannose was abolished, to our surprise, C3 epimerization of GDP-4-keto-*manno*-heptose was retained. To the best of our knowledge, this is the first example where both C3 and C5 reactions do not rely on the same catalytic histidine to initiate catalysis by deprotonation. DdahB, which lacks significant C5 epimerization activity, likewise retained C3 epimerization of GDP-4-keto-*manno*-heptose but not GDP-4-keto-mannose. We have not been able to identify any alternative residue at the active site of MlghB or DdahB that could function as the catalytic base.

We were also surprised by the differences of effects of Y132F and Y134F mutations in MlghB and DdahB. Although the Y134F mutation abolished all activity of MlghB on heptose as expected, significant activity was observed on heptose for DdahB and on mannose for the Y134F mutants of both enzymes. The residual activity points to the presence of an alternative proton donor. Nearby candidate Y132 influenced catalysis mostly in MlghB, with drastic reduction of catalysis in the Y132F mutant and abolished catalysis in the Y132F/Y134F double mutant. In contrast, the Y132F mutation had little impact on catalysis by DdahB and the double-mutant Y132F/Y134F was still active. Thus, Y132 was less important in DdahB than in MlghB although Y132F occupied the same position in both enzymes and in RmlC. We note these results contradict several other studies on hexose substrates. We can only suggest that the larger active site, necessary to process the C7 substrate, allows water molecules to enter the active site and mediate proton abstraction and donation.

Given the similarity of DdahB and MlghB, we were surprised that DdahB mainly epimerized the C3 position. Other RmlC-like monoepimerases have been structurally characterized, including the dTDP-4-keto-6-deoxy-d-glucose-5-epimerase (EvaD) (31) and the dTDP-4-keto-6-deoxy-glucose-3-epimerase (32). An EvaD study proposed that the C5 activity and lack of C3 epimerization of EvaD arose from a difference in the orientation of the catalytic Tyr with respect to that of dTDP-sugar–bound complex of RmlC (31). This reorientation was proposed to arise from other sequence changes. The study of the C3 epimerase, dTDP-4-keto-6-deoxy-glucose-3-epimerase, concluded that the position of the Tyr was not critical to whether the enzyme was a C3, C5, or a monoepimerase; however, it did not identify a molecular basis for mono-epimerization. Because the catalytic Tyr 132 in MlghB and DdahB has the same conformation in both structures, we do not favor the position of Tyr 132 as the key factor.

Comparison of DdahB and MlghB identified a difference in the conformation of the C-terminus. This region contains the predicted catalytic Asp 173 residue (by analogy to RmlC). The change in C-terminus is caused by Glu 171 in DdahB (found as Asp 171 in MlghB) that forms a hydrogen bond that stabilizes an alternative conformation of the C-terminus, which located the Asp 173 out of position for a catalytic role. Given that DdahB is active, it seems unlikely that its C-terminus is permanently in this conformation. Rather, we suggest that by accessing this conformation, DdahB compromises its catalytic efficiency and this compromise in catalytic efficiency may underpin the apparent C3 mono-epimerization.

Despite the sequence similarity to GFS and GME, we found no evidence that either DdahC or MlghC can act as epimerases. Given the assays we used, were such activity present, we would have detected it. Thus, both DdahC and MlghC are reductases devoid of epimerase activity and although in sequence terms less similar to reductase RmlD, functionally they are in fact RmlD homologues.

The study of these reductases was complicated by the fact the epimerases generate an equilibrium of substrates, and the instability of the 4-keto-intermediates precludes their individual purification. Thus, our assays have a mixture of potential substrates and therefore multiple potential products. When incubated with the mix of mono (C3 or C5) or double (C3, C5) epimers of GDP-*manno*-heptose created by MlghB, MlghC reduced exclusively the double-epimerized product. Thus, MlghC enzyme acts to ensure that only one product is made in the natural pathway. Given this same mixture of compounds, DdahC is also specific for one GDP-*manno*-heptose–derived epimer, but this enzyme processes only the mono-C3–epimerized variant, which is produced by DdahB in the natural pathway. Thus, these enzymes are able to distinguish between substrates that differ only in their chirality at C5.

Given the specificity observed for the C7 sugar substrates, we investigated the behavior of the enzymes with C6 substrates. To our surprise, both reductases were able to reduce unepimerized GDP-4-keto-mannulose, although neither enzyme can reduce unepimerized GDP-4-keto-*manno*-heptulose. The enzymes were also able to reduce the mono-epimerized GDP-4-keto-mannulose (which we suggest has occurred at C3). Although this was expected for DdahC, which reduces the C3-epimerized C7 substrate, it was surprising that MlghC was able to do this. Because multiple C6 substrates with different structures can be processed, the active site must have sufficient space to allow quite different C6 molecules to be correctly positioned for hydride transfer. This flexibility is not observed with C7 substrates. Our analysis would suggest that MlghC is more tolerant than DdahC because it gives rise to a third product that we have not been able to definitively identify. Structural comparison reveals that the active site of the heptose specific enzymes is notably more open than homologues that process hexose substrates. We were able to abrogate the enzyme activity against C7 substrate but retain C6 activity by making a double-mutant T110Y/H180R in DdahC, which was designed to reduce the volume at the active site. Because the double mutant also mimicked the MlghC dyad, a switch of heptose epimer specificity from P4α to P4γ was expected, but instead, all heptose activity was lost. Interestingly, the H180R mutation on its own has no effect on heptose catalysis and increasing the volume at the active site by making the T110C increased catalytic efficiency without changing epimer specificity. The opposite was observed on mannose, with no effect of T110C but enhanced catalysis of P1’ in H180R. The introduction of T110C mutation leads to the formation of a GFS H/C dyad, but this did not lead to epimerization, consistent with the general observation that it is hard to introduce a new activity into an enzyme by simple mutagenesis. Epimerization in GFS requires a precisely positioned catalytic dyad and a much faster rate of epimerization compared with the rate of reduction of the nonepimerized substrate. It seems likely that the C7 pathways in *Campylobacter* have evolved from the C6 RmlC/RmlD like enzyme pairing, and thus, there has been little evolutionary pressure to evolve the reductase into a combined epimerase reductase enzyme.

*In vivo*, the reductase enzymes are not thought to be involved in mannose-utilizing pathways, and the rates for the hexose compounds are indeed much slower than those for the heptose substrates. The existence of heptose- *versus* hexose-specific enzymes may also allow full pathway segregation and independent regulation. It may be that these predominantly heptose-utilizing enzymes could partially compensate for deletion or antibiotic inhibition of the normal hexose enzymes. Furthermore, the ability to reduce a range of C6 keto sugars may have value in bioengineering.

In conclusion, we have analyzed the function, mechanism, and structure of two pairs of *C. jejuni* enzymes that are conserved across *Campylobacters* and are responsible for the production of two key heptose sugars. Our work has elucidated the factors that underpin their specificity for the heptose rather than hexose. We also discovered that the enzymes possess some ability to process the smaller hexose substrates consistent with a view that enzymes have evolved from the more common hexose system. The heptose sugars are critical components of the capsular polysaccharide that protects the bacteria from the host and allows the bacteria to colonize the gut. Economically, the most important host is the chicken as most cases of human campylobacteriosis in developed countries are linked with consumption of undercooked contaminated chicken meat. Our end goal is to develop an inhibitor specifically against the enzymes of heptose modification pathways in *C. jejuni* and that could be used to treat or prevent colonization of chicken by *C. jejuni*. Decreased levels of *C. jejuni* colonization of chicken will reduce human gastrointestinal illness. Our enzymes can be used for high-throughput screens for inhibitors, and our structural biology data may allow optimizing the inhibitor hits identified.

## Experimental procedures

### Protein expression and purifications for enzymology

All Mlgh and Ddah enzymes from *C. jejuni* strains 81 to 176 and NCTC-11168, respectively, were produced with noncleavable histidine tags using our pET constructs and BL21(DE3)pLysS (MlghB, DdahA, and DdahB) or ER2566 (MlghC and DdahC) *E. coli* for expression as described (3). HP0044 was produced as a GST-tagged protein from pGEX-2T with expression in DH5α as described (1). The proteins were analyzed by SDS-PAGE with Coomassie staining (Fig. S9), their concentration determined by Bradford assay, and all were frozen in the presence of 25% glycerol at −20 °C.

### Structural modeling

Structural models for the epimerases and reductases were generated using the SWISS-MODEL workspace (http://swissmodel.expasy.org/) (33) in automatic mode. The best model was chosen based on the QMEAN score (34): dTDP-6-deoxy-D-xylo-4-hexulose C3/C5 epimerase (a.k.a. RmlC) from *S. enterica* (PDB code: 1DZR, 2.17-Å resolution (15)) for the epimerases and GDP-4-keto-6 deoxy-mannose C3/C5 epimerase, C4 reductase (a.k.a. GFS) from *E. coli* (PDB code: 1BSV, 2.20-Å resolution (21)) for the reductases. The modeled 3D structures were visualized and analyzed using PyMol software (https://www.pymol.org/).

### Site-directed mutagenesis

Site-directed mutagenesis was performed using the QuikChange method following manufacturer’s instructions (Stratagene) using *E. coli* DH5*α* cells. The primers used for mutagenesis are listed in Table S6. The entire gene was sequenced for each mutated enzyme. DNA sequencing was carried out at the Robarts Institute sequencing facility (London, Ontario, Canada).

### Capillary electrophoresis analysis

The capillary electrophoresis (CE) analyses were as described (1), using fused silica capillary of 75-μm inner diameter on a P/ACE MDQ instrument with the 32 Karat software. After injection of the sample at 0.5 psi for 4 s, separation was performed using 25-mM Borax, pH 9, and 26 kV for 20 min, with detection at 254 nm. Because electrophoretic mobility in CE changes over time (because of ion transfer between electrodes at each run), NAD(P) and/or NAD(P)H were included in most reactions even when not needed for catalysis, to serve as internal standards facilitating alignments of reaction peaks on CE traces. Reactions performed with mutated *versus* WT enzyme or with Mlgh *versus* Ddah enzymes were also coinjected to ascertain if the peaks were the same or not. When applicable, peak areas were integrated using the 32 Karat software and statistics were performed using T-tests.

### Enzymatic assays

The GDP-D-glycero-D-*manno*-heptose substrate was prepared as described (1), whereas GDP-mannose was purchased from Sigma. All reactions were performed in 200-mM Tris HCl, pH 7.5 to 8, in the presence of 0.1- to 0.5-mM substrate and 0.37- to 0.5-mM NADPH/NADP^+^ mix (40/60 %/%) for a total volume of 10 μl. All were incubated at 37 °C and frozen at −20 °C until processed for CE analyses. For all time course experiments, a master reaction was set up and aliquoted before incubation for the allocated time. To generate equimolar stocks of heptose-based and mannose-based substrates to compare the substrate specificity of DdahB and MlghB (Fig. 2), reactions of 0.54-nmol DdahA and 26-nmol GDP-*manno*-heptose were incubated for 20 min and yielded 100% conversion into the 6-deoxy-4-keto derivative (=26 nmol) and reactions of 0.95-nmol DdahA and 55-nmol GDP-mannose were incubated for 90 min and yielded 40% conversion into the 6-deoxy-4-keto derivative (=22 nmol). DdahA was eliminated by ultrafiltration (10 kDa cut-off). Eleven nmol of each 6-deoxy-4-keto derivative was then reacted in parallel with 0.1 nmol of MlghB or DdahB, and aliquots were withdrawn over time for CE analysis. For the analysis of the substrate specificity of MlghC and DdahC (Fig. 7), 58-nmol GDP-*manno*-heptose were incubated with 0.57 nmol of DdahA and 0.18-nmol MlghB for 30 min, which yielded ∼63% substrate conversion split into 6-nmol P4α, 11.8-nmol P4β, and 18.7-nmol P4γ. Reactions of 144-nmol GDP-mannose, 1.6-nmol DdahA, and 0.18-nmol MlghB were incubated for 2 h and yielded 15% substrate conversion into P4’ (21.6 nmol). DdahA and MlghB were eliminated by ultrafiltration (10 kDa cut-off). Half of each reaction was supplemented with 0.12 nmol of MlghC or DdahC along with NADPH (16 nmol), incubated at 37 °C, and aliquots were withdrawn over time for CE analysis.

For other experiments, stoechiometries were adapted on an ad hoc basis to account for the need to produce enough substrate for the enzyme under study and for the efficacy of said enzyme. In some cases, the design also accounted for the need to eliminate most mannose by C4, C6 dehydration before addition of the reductases whose reaction product PII overlaps with the mannose peak and complicates data analysis. Specifics are provided in the legends to the figures.

### Cloning of target genes for structural characterization

All primers used are listed in Table S7. The *ddahB* and *mlghB* genes were subcloned from the pET derivative above into pEHISTEV to introduce an N-terminal TEV protease-cleavable His_6_ tag (35). The vector and the PCR-amplified genes were digested with NcoI and BamHI except for *ddahB* that was digested with AflIII and BamHI. For structural characterization, the Met-Ser linker between the TEV protease site and the target sequence of *ddahB* in the pEHisTEV vector was removed by PCR, following standard procedures of Quik-Change site-directed mutagenesis (36). Also, a TEV protease site was inserted between the N-terminal His_6_ tag and the *ddahC* and *mlghC* sequences in the pET23 vector derivative (3, 4) by site-directed mutagenesis (36).

### Structural biology

All constructs were expressed in *E. coli* C43 (DE3) cells using LB growth medium. Expression was induced with 0.5- to 1-mM IPTG once absorbance at 600 nm reached 0.4 to 0.6 and carried out at 25 °C for 20 h except for noncleavable DdahC (37 °C, 3 h). Pellets of *E. coli* expressing TEV-cleavable DdahB, DdahC, MlghB, and MlghC were resuspended in PBS. Pellets of *E. coli* expressing non-TEV–cleavable DdahC and MlghC were resuspended in 10-mM sodium phosphate, 500-mM NaCl, 10-mM imidazole, pH 7.5. 1-mg DNAse was added to the resuspended cells, which were then lysed using a cell disrupter at 30 kpsi at 4 °C (Constant Systems), followed by centrifugation at 18,000 rpm 20 min at 4 °C using a JA 25.50 rotor in a J-26XP centrifuge (Beckman Coulter). The supernatants were filtered using a 0.45-μm filter. Target proteins were purified by nickel affinity chromatography using standard procedures (35), except the columns were performed in batch with 1-h binding at 4 °C for all TEV-cleavable enzymes and 30 min at room temperature (RT) for non-TEV–cleavable enzymes. Target proteins were eluted with the lysis buffer plus 400-mM imidazole, pH 7.5. TEV-cleavable proteins were dialyzed overnight at RT with His-tagged TEV protease in 50-mM Tris, pH 7.5, 400-mM NaCl (500-mM NaCl for DdahC and MlghC). The nickel affinity purification step was repeated to remove the non-TEV–cleaved protein and TEV protease; this time, washing with the dialysis buffer to remove nonspecifically bound protein. The proteins were then concentrated using a Vivaspin 20 concentrator at 4 °C, and once the target volume was reached, the sample was centrifuged for 10 min at 20,817*g*. Proteins were then loaded onto Hiload 16/60 Superdex 200 pg or Superdex 200 10/300 GL columns in 10-mM Tris, pH 7.5, and 150-mM NaCl using the Bio-Rad NGC chromatography system. Non-TEV–cleavable proteins were concentrated and purified by size-exclusion chromatography, as described above, directly after the nickel affinity chromatography step. Fractions containing the target protein, as determined by SDS-PAGE, were pooled and concentrated as required. The identity and integrity of the purified proteins were confirmed by mass spectrometry (Biomedical Sciences Research Complex (BSRC) Mass Spectrometry and Proteomics facility, the University of St Andrews).

For SEC-MALS analysis, a Dawn Heleos II light scattering detector (Wyatt) and an Optilab T-rEX dRI detector (Wyatt) were connected downstream to a Bio-Rad NGC chromatography system for measurement after size-exclusion chromatography. Before use, all machines were equilibrated in the size-exclusion chromatography buffer until the baselines had stabilized. Data were processed using the ASTRA software package (Wyatt).

Protein crystallization carried out using sitting-drop vapor diffusion trials was set up at RT 96-well Intelli-plates using the Gryphon Crystallization Robot (Art Robbins Instruments). DdahB, MlghC, and DdahC crystallization trials were set up at 11 mg/ml and MlghB crystallization trials were set up at 12 mg/ml. DdahB crystallized in JCSG-plus (Molecular Dimensions) well D1 (24 % (w/v) PEG 1500, 20% (v/v) glycerol), MlghB crystallized in an in-house screen St Andrews PEG 1 well E3 (31.05% (w/v) PEG 1500, 0.25 M sodium-potassium phosphate, 3.83% (v/v) 1,4-dioxane), DdahC crystallized in 54% (w/v) PEG 400, 0.1 M Hepes, pH 7.5, 0.08 M ammonium citrate, and MlghC crystallized in 24.63% (w/v) PEG 8000, 0.1 M Bicine, pH 8.5, and 0.12 M sodium citrate 0.05% (w/v).

For complexes, crystals of DdahB were soaked overnight at 20 °C in mother liquor supplemented with 5-mM GDP-mannose (Sigma Aldrich), and MlghB was incubated with 20-mM GDP-mannose before crystallization.

All crystals were flash-frozen directly from the well or supplemented with 20% glycerol in liquid N2 before data collection, and all but DdahC were collected at Diamond Light Source (IO4-1) and processed with XIA2 pipeline (37). DdahC X-ray diffraction data were collected using a Rigaku 007HFM rotating copper anode X-ray generator and Saturn 944 charge-coupled device detector and processed using iMosflm (38), Pointless (39), and Aimless (40).

DdahB and DdahC crystal structure was solved using CCP4 online (41). MlghB and MlghC were solved using Phaser (42) with the DdahB/C enzymes as search models. Models were completed by manual building in Coot (43), followed by refinement conducted using REFMAC5 (44). Appropriate translation-libration-screw restraints for refinement were determined using the TLSMD server (45) and used in refinement using REFMAC5 (44). The native oligomeric state, interfaces, and assembly of all structures were determined using PDBePISA ('Protein interfaces, surfaces and assemblies') service PISA at the European Bioinformatics Institute (http://www.ebi.ac.uk/pdbe/prot_int/pistart.html, (24, 46)).

## Data availability

All enzymology data described in this article are presented in the article and in the supplementary information section. Beyond the structural information described in this article, structural data have been deposited in PDB and the accession numbers for all structures are indicated in Tables 1 and 3.

## Conflict of interest

The authors declare that they have no conflicts of interest with the contents of this article.
