# Supplementary material for: Structure–function studies of the C3/C5 epimerases and C4 reductases of the Campylobacter jejuni capsular heptose modification pathways
Source: J Biol Chem. 2021 Jan 30;296:100352. doi: 10.1016/j.jbc.2021.100352 (PMC7949155; doi:10.1016/j.jbc.2021.100352)

Structure / function studies of C3/C5 epimerases and C4 reductases of the *Campylobacter jejuni* capsular heptose modification pathways.

Heba Barnawi[+1], Laura Woodward[+2],Natalie Fava[1], Mikhail Roubakha[1], Steve D. Shaw[1], Chelsea Kubinec[1], James H. Naismith[2,3,4]* and Carole Creuzenet[1]*

Supplementary information

Tables:

Table S1: Comparative functional analysis of the conserved residues in the epimerases binding site.

Table S2: Comparative functional analysis of the conserved residues in the reductases binding site.

Table S3: SEC-MALS data summary.

Table S4: Summary of root mean square deviation (rmsd) data to compare the structural similarity of the crystallised epimerases with one another and with RmlC.

Table S5: Summary of root mean square deviation (rmsd) data to compare the structural similarity of the crystallised reductases with one another and with previously crystallized enzymes.

Table S6: Primers used for site-directed mutagenesis.

Table S7: Primers used to prepare expression constructs for crystallography.

Figures:

Figure S1: SEC-MALS traces for A) DdahB, B) MlghB, C) DdahC, and D) MlghC.

Figure S2: Superimposition of the DdahB and MlghB dimers along with GDP and GDP-mannose.

Figure S3: Quantitation of catalysis for MlghB and DdahB N121S and H67A/N mutants. Panels A and B.

Figure S4: Effect of D173A mutation on heptose catalysis in DdahB and MlghB.

Figure S5: Kinetics of catalysis for MlghB Y134F, Y132F and double mutant.

Figure S6: Effect of Y132F, Y134F and YY/FF double mutation on heptose catalysis in DdahB.

Figure S7: Superimposition of the MlghC (orange) and DdahC (blue) monomers.

Figure S8: Superposition of MlghC (orange) with human GDP-L-fucose synthase (GFS) (grey).

Figure S9: Catalytic activity of mutated DdahC reductase.

Figure S10: Representative examples of Coomassie-stained SDS-PAGE gels for purified proteins diluted to equal concentrations for use in kinetic analyses.

Table S1: Comparative functional analysis of the conserved residues in the epimerases binding site. Underlined residues were targeted by site-directed mutagenesis in DdahB and/or MlghB. *St. suis*: *Streptococcus suis*. *S. ent: Salmonella enterica*.

<table>
<tr><th>RmlC
St. suis</th><th>Function (31)</th><th>RmlC
S. ent.</th><th>Function (30,32)</th><th>DdahB
MlghB</th><th>Predicted function</th></tr>
<tr><td>H76</td><td>Catalytic base for epimerization at C3 and C5</td><td>H63</td><td>Catalytic base for epimerization at C3 and C5</td><td><u>H67</u></td><td>Catalytic for epimerization at C3 and C5</td></tr>
<tr><td>D180</td><td>His76-Asp dyad. Increases basicity of H76</td><td>D170</td><td>His63-Asp dyad. Increases basicity of H63</td><td><u>D173</u></td><td>Catalytic His67-Asp dyad.</td></tr>
<tr><td>Y140</td><td>Catalytic. OH group is 3.7Å from C3 and 3.5Å from C5.</td><td>Y133</td><td>Catalytic. Proton donor for epimerization at C5. Not essential at C3</td><td><u>Y134</u></td><td>Catalytic for epimerization at C3 and C5</td></tr>
<tr><td>K82</td><td>Catalytic. Stabilizes the negative charge after deprotonation</td><td>K73</td><td>Catalytic for epimerization at C3</td><td><u>K74</u></td><td>Catalytic for epimerization at C3</td></tr>
<tr><td>F36</td><td rowspan="3">Interacts with thymidine ring.</td><td>F27</td><td rowspan="3">Interacts with thymidine ring.</td><td>I31</td><td rowspan="3">Interacts with guanidine ring.</td></tr>
<tr><td>Y145</td><td>Y139</td><td>Y142</td></tr>
<tr><td>N63</td><td>N50</td><td>N57 in MlghB, H57 in DdahB</td></tr>
<tr><td>H29</td><td>Stacks against the ribose ring.</td><td>F20</td><td>Stacks against the ribose ring.</td><td>F24</td><td>Stacks against the ribose ring.</td></tr>
<tr><td>N127</td><td>Binds O4 (substrate recognition)</td><td>H120</td><td>Unknown. Could interact with O4</td><td><u>N121</u></td><td>Binds O4 (substrate recognition)</td></tr>
<tr><td>F129</td><td rowspan="2">Van der Waals interactions with C6 and O6. Not conserved in all RmlC</td><td>F122</td><td>Unknown</td><td>H123</td><td>Interacts with the sugar ring</td></tr>
<tr><td>Y138</td><td>F131</td><td>Unknown</td><td><u>Y132</u></td><td>Catalytic</td></tr>
<tr><td>G94</td><td>Unknown</td><td>D84</td><td>His120-Asp dyad. Unknown function</td><td>Q85</td><td>Catalytic. May interact with N121.</td></tr>
</table>

Table S2: Comparative functional analysis of the conserved residues in the reductases binding site: Underlined residues were targeted by site-directed mutagenesis in DdahC and/or MlghC.
Na: Not applicable

**GFS** *E. coli* **(34)**	**RmlD** *S. ent* **(44)**	**Function**	**DdahC**	**MlghC**	**Predicted function**
**S107**	**T104**	Catalytic triad for reduction at C4	**S108**	**S107**	Catalytic triad. M110 potentially serving instead of F136 in MlghB based on structure.
**Y136**	**Y128**		**Y137**	**F136**	
**K140**	**K132?**		**K141**	**K140**	
**C109**	**Na or Y106**	General acid / base dyad for epimerization at C3 and C5 in GFS.	**T110**	**Y109**	Catalytic
**H179**	**Na**		**H180**	**R179**	
**Na**	**Y106**	Interaction with rhamnose moiety in RmlD	**T110**	**Y109**	Substrate specificity
**Na**	**R260**	Hydrogen bonding to the pyrophosphate moiety of dTDP-L-rhamnose	**H180**	**R179**	Hydrogen bonding to pyrophosphate of GDP.
**S108**	**D105**	Predicted to interact with the sugar ring	**T109**	**G108**	Interact with the sugar ring
**N165**	**W153**		**N166**	**N165**	
**L184**	**M166**	Hydrophobic pocket for guanidine ring (GFS) or dTDP (RmlD) moiety of nucleotide	**I185**	**L184**	Hydrophobic pocket for guanidine ring of nucleotide
**V201**	**S177**		**I1231**	**I230**	
**V180**	**Na**		**V181**	**V180**	
**W202**	**V178**		**W232**	**W231**	
**G67**	**A66**	Unknown	**G68**	**C68**	Important for binding co-factor?

Table S3: SEC-MALS data summary.

a Mn is defined as: $\Sigma N_i M_i^2 / \Sigma N_i M_i$, where M_i corresponds to the molecular weight of the protein and N_i corresponds to the number of proteins of that molecular weight.
b Mw is defined as: $\Sigma N_i M_i / \Sigma N_i$.
c Polydispersity is the range of molecular weights occupied by the sample and is defined as Mw/Mn, with 1.0 corresponding to a monodisperse sample.
d Data for non-TEV-cleavable DdahC and MlghC are shown.

	**DdahB**	**MlghB**	**DdahC**[d]	**MlghC**[d]
**Molar mass moments (g/mol)**				
Mn[a]	4.1×10^4 (± 0.4%)	4.2×10^4 (± 3.4%)	9.7×10^4 (± 0.7%)	7.9×10^4 (± 0.4%)
Mw[b]	4.1×10^4 (± 0.4%)	4.2×10^4 (± 3.5%)	9.8×10^4 (± 0.7%)	8.0×10^4 (± 0.4%)
**Polydispersity**[c]				
Mw/Mn	1.0 (± 0.6%)	1.0 (± 4.9%)	1.0 (± 1.0%)	1.0 (± 0.6%)

Table S4: Summary of root mean square deviation (rmsd) data to compare the structural similarity of the crystallised epimerases with one another and with RmlC. nd: not determined

**Enzymes compared**	**MlghB**	**DdahB**	**MlghB + GDP-mannose**	**DdahB + GDP-mannose**
**MlghB**	-	0.7 Å over 163 residues	0.4 Å	Nd
**DdahB**	0.7 Å over 163 residues	-	nd	0.24 Å
**RmlC *S. ent***	1.7 Å over 164 residues	1.5 Å over 154 residues	nd	Nd

Table S5: Summary of root mean square deviation (rmsd) data to compare the structural similarity of the crystallised reductases with one another and with previously crystallized enzymes.

**Enzymes compared**	**MlghC**	**DdahC**
**MlghC**	-	1.3 Å over 333 residues
**DdahC**	1.3 Å over 333 residues	-
**GMER *E. coli***	1.9 Å over 315 residues	2.0 Å over 315 residues
**GFS *E. coli***	2.1 Å over 265 residues	2.2 Å over 271 residues
**RmlD *S. ent***	2.3 Å over 253 residues	2.84 Å over 263 residues
**GFS human + $NADP^+$ and GDP-L-Fucose**	2.4 Å over 285 residues	2.9 Å over 289 residues

Table S6: Primers used for site-directed mutagenesis. Only forward primer sequences are indicated. Reverse primers have the exact complementary sequences. Codons for mutated residues are in bold and the mutated nucleotides are underlined.

[a] DdahB and MlghB Y132F/Y134F mutants were generated using the single mutants Y134F as a template.

[b] DdahC H180R/T110Y was made using the single mutant H180R as a template.

Protein	Mutation	Primers Sequence (5' – 3')
DdahB	N121S	GTGCCAGCAGGTTTTGGA**AGC**GCTCATTATGTTACTAGTG
	H67A	CAATGTTATTCGCGGTATC**GCT**GGTGATGTAAAAACTTATAAGCTTG
	H67N	CAATGTTATTCGCGGTATC**AAT**GGTGATGTAAAAACTTATAAGCTTG
	K74A	CCATGGCGATGTAAAAACTTAT**GCG**CTTGCAACTTG TGTTTATGG
	Y134F	GTTACTAGTGAAAGTGCAGTTTATTAC**TTT**AAATGTGCTTATAAAGGAGATTATGTG
	Y132F	GTTACTAGTGAAAGTGCAGTT**TTT**TACTATAAATGTGCTTATAAAG.
	Y132F / Y134F[a]	GTTACTAGTGAAAGTGCAGTT**TTT**TAC**TTT**AAATGTGCTTATAAAG.
MlghB	N121S	ATTACCACCAAATATGGGA**AGC**TCTCATTATGTGAG TTCAAAG
	H67A	CCCATTTTAATGTTTTACGTGGAAT**AGC**CGGAGATGTGAAAACTTAC
	H67N	CCCATTTTAATGTTTTACGTGG**AAT**AAACGGAGATGTGAAAACTTAC
	Y134F	CAAAGGAAGCAGTGTATTAT**TTT**AAACTTGCTTATGAGGGG
	Y132F	GAGTTCAAAGGAAGCAGTG**TTT**TATTATAAACTTGCTTATG
	Y132F / Y134F[a]	GAGTTCAAAGGAAGCAGTG**TTT**TATTTTAAACTTGCTTATG
	K74A	CACGGAGATGTGAAAACTTA**CGC**ACTTGTAACTTGTGTCTATGGAG
DdahC	H180R	GACAAATTTGATCTTGAAAAATCT**CGT**GTATTGCCTGGAATTTTAAGAAAAAATG
	T110Y	GCTACTTTTCATAGCTTCAACT**TAC**GTTTATCCTAAAAATGCAACATTG
	H180R / T110Y[b]	GCTACTTTTCATAGCTTCAACT**TAC**GTTTATCCTAAAAATGCAACATTG
	T110C	CTACTTTTCATAGCTTCAACT**TGC**GTTTATCCTAAAAATGCAACATTG
MlghC	C68A	GACTGCAGTCTTACCT**GCT**GGTGCTGCAAATGTCG

Table S7: Primers used to prepare expression constructs for crystallography.

Enzyme *gene*	Cloning step	Primer name	Primer sequence
DdahB *cjj1430*	Subcloning	CJPG1430cP3	CTCACATGTCCATGGCAATAGAATTTAATATAC
		CjPG1430cP2	GCTGGATCCTTATCCTTTATTTTTAGTTGCT
DdahB *cjj1430*	Linker removal	DdahB MS linker forward	GGGCGCCATGGCAATAGAATTTAATATACAAGAATCAAAAA
		DdahB MS linker reverse	TATTGCCATGGCGCCCTGAAAATACAGGTTTTCGGTCG
MlghB *cj1430*	Subcloning	CJ1430P2	AGGGTCCATGGCAATAGAATTTGATATA
		CJ1430P3	GCGTCGGATCCTTATCCTTTATTTTTAGTTGCAA
DdahC *cjj1427*	TEV insertion	TEV-DdahC forward	GAAAACCTGTATTTTCAGGGCATGCAAAAAGATTCTAAAATTT
		TEV-DdahC reverse	CCTGAAAATACAGGTTTTCGCCCATGGAACCGTGGTGG
MlghC *cj1428*	TEV insertion	TEV-MlghC forward	GAAAACCTGTATTTTCAGGGCATGCAAACAAATTCAAAAATAT
		TEV-MlghC reverse	CCGAAAATACAGGTTTTCGCCCATGGAACCGTGGTGG

Figure S1: SEC-MALS traces for A) DdahB, B) MlghB, C) DdahC, and D) MlghC. For both DdahC and MlghC, the results are for the non-TEV-cleavable constructs. The change in light scattering over time is shown in red, the change in refractive index over time is shown in blue, the change in UV over time is shown in green (shown in B only) and the change in molar mass (g/mol) over time is shown in black.

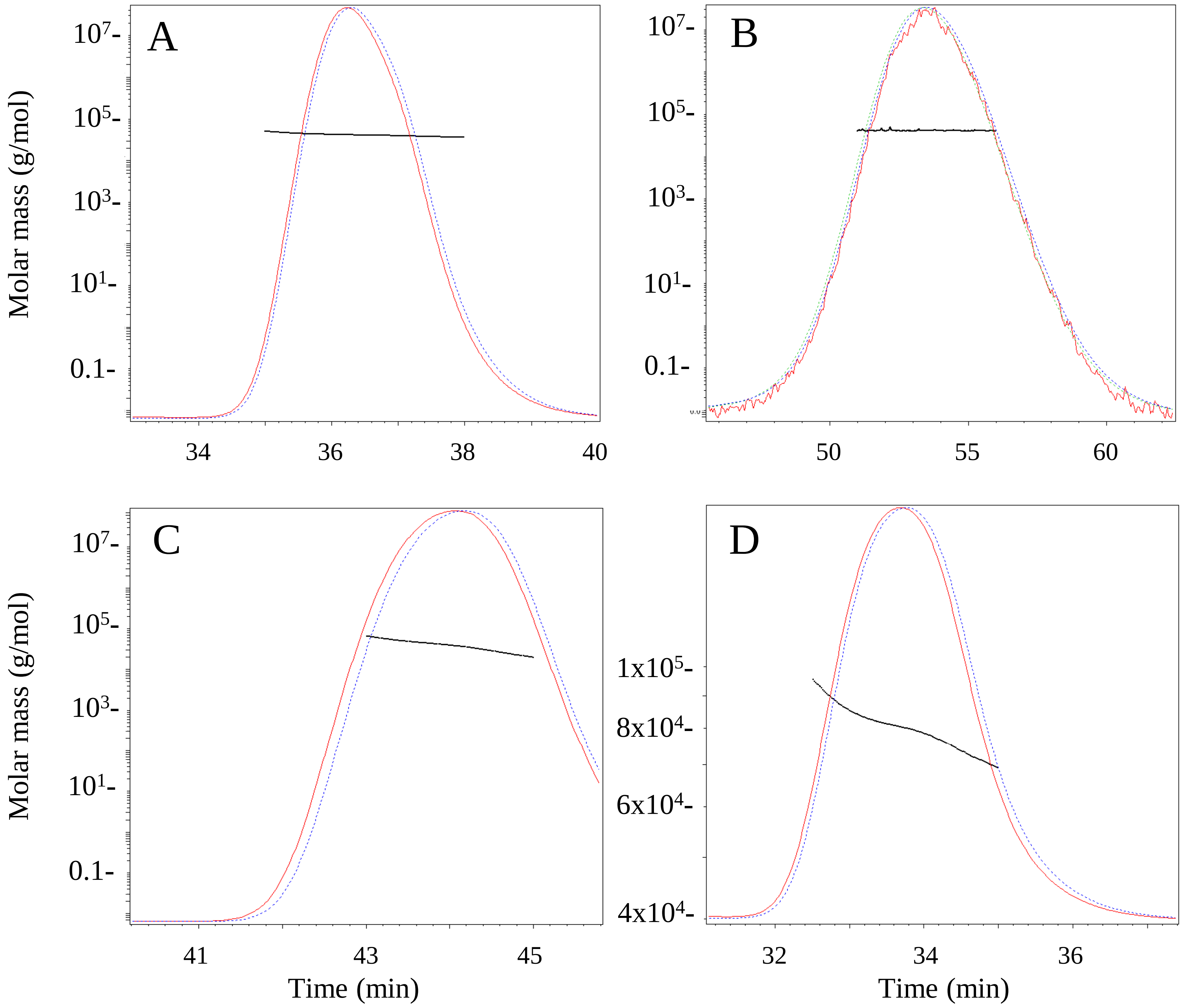

Figure S2: Superimposition of the DdahB and MlghB dimers along with GDP and GDP-mannose. The DdahB dimer shown again in yellow and pink, superimposed with MlghB which is shown in green and blue. The structures are essentially identical, GDP molecules are located in identical positions relative to the secondary structure of both proteins.

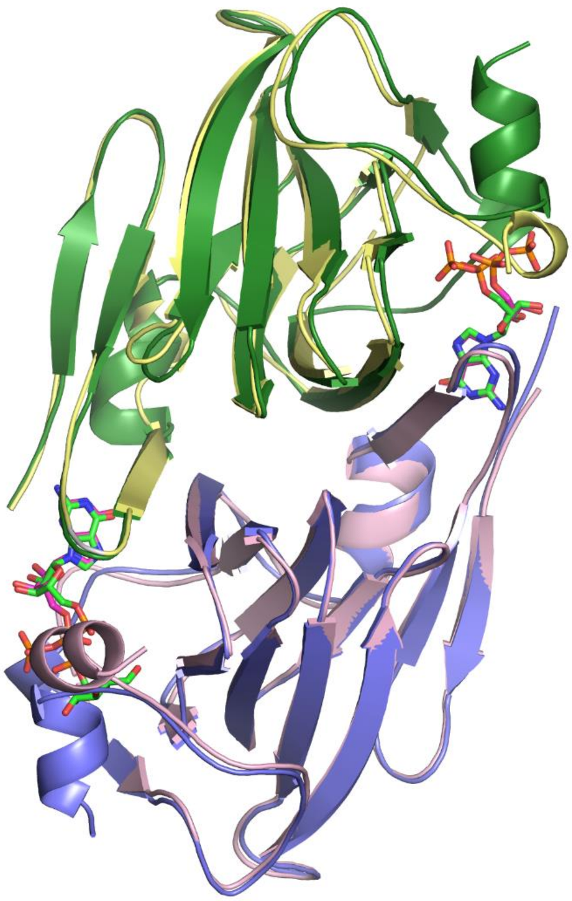

Figure S3: Quantitation of catalysis for MlghB and DdahB N121S and H67A/N. Panels A and B. Activity on heptose for DdahB and MlghB. **Panel C.** Activity on mannose for MlghB. DdahB mutants were inactive on mannose, thus no panel is available. Histograms are derived from data shown on Figure 4 (30 min reactions). For kinetics, reactions contained 0.17 mM of heptose and 0.1 μM of epimerase, or 0.77 mM mannose (yielding ~ 0.4 mM of P1' substrate) and 0.1 μM of MlghB. Data in % of all substrate. * $p<0.01$. ** $p<0.005$.

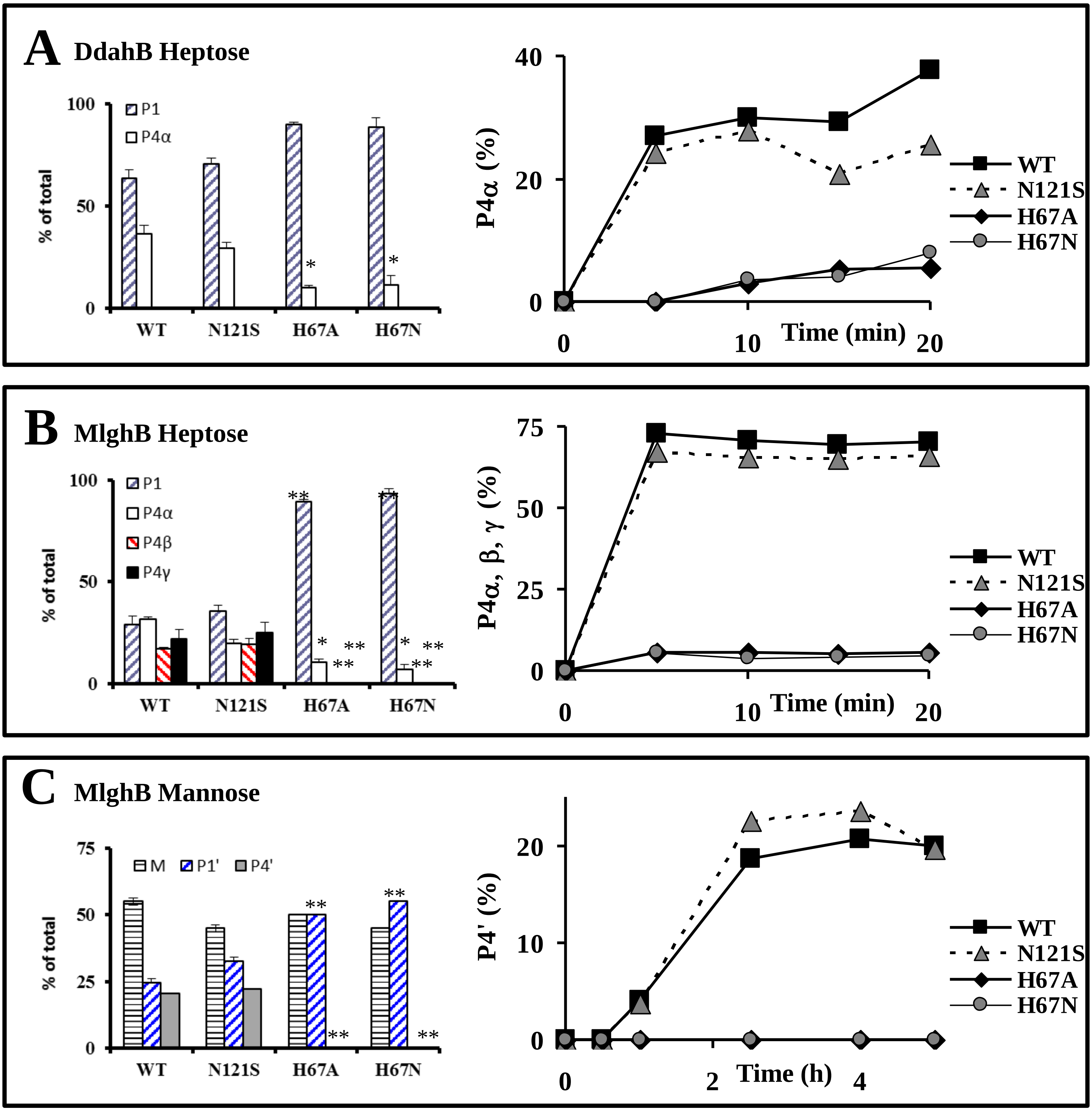

Figure S4: Effect of D173A mutation on heptose catalysis in DdahB and MlghB. the tests were done at 2 different enzyme dilutions (5 fold differences between diluted and non diluted series) and 2 different incubation times. Apart from these modifications, all reaction conditions are as described in Figure 5A. Each bar represents the average quantitation from 2 independent batches of purified enzymes. This complements the data shown in Figure 5A.

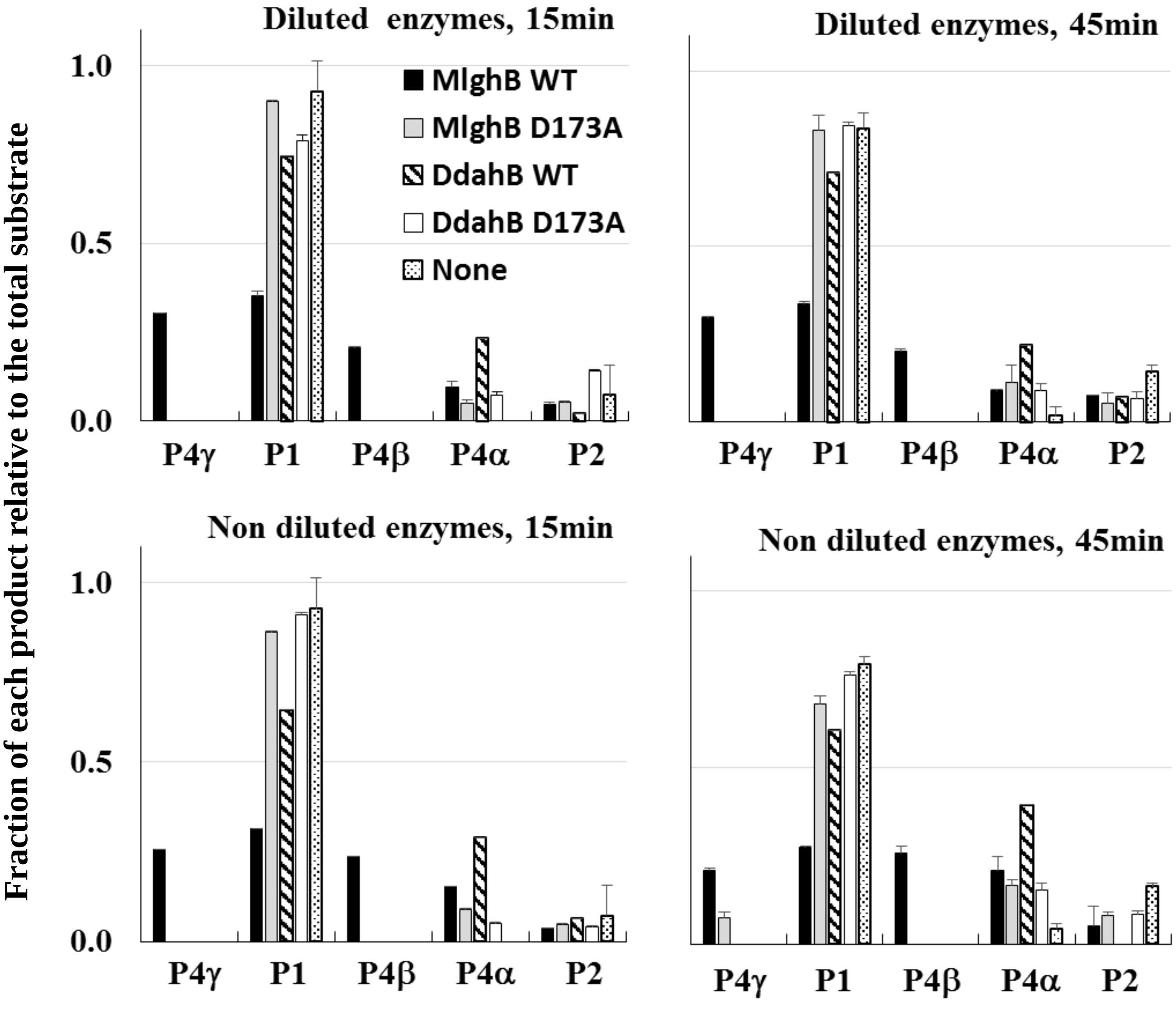

Figure S5: Kinetics of catalysis for MlghB Y134F, Y132F and double mutant. Panel A: Activity on heptose. **Panel B.** Activity on mannose. Reaction conditions are as described in Figure 6.

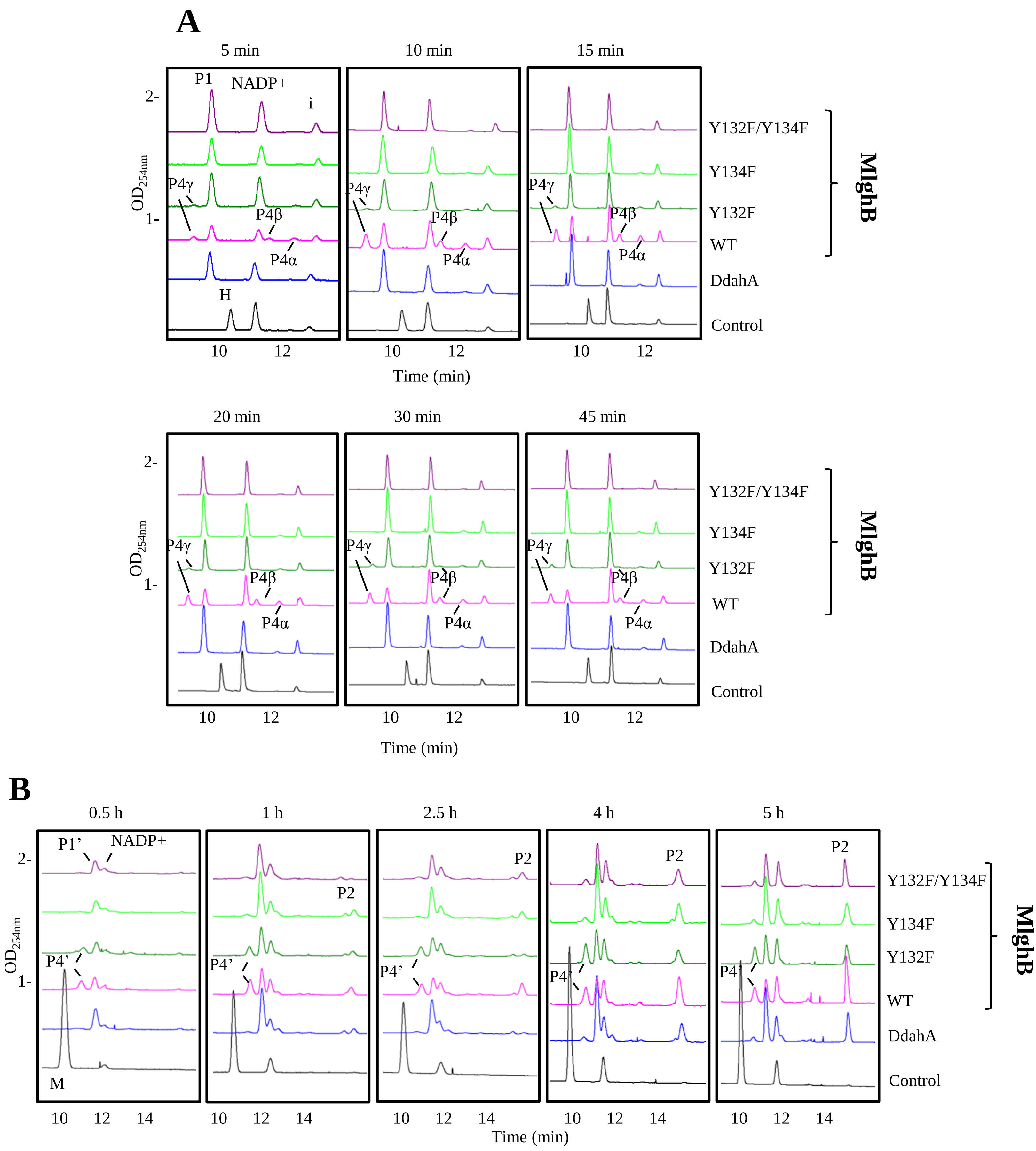

Figure S6: Effect of Y132F, Y134F and YY/FF double mutation on heptose catalysis in DdahB. The tests were done at 2 different enzyme dilutions (5 fold differences between diluted and non diluted series) and 2 different incubation times. Apart from these modifications, all reaction conditions are as described in Figure 5. Each bar represents the average quantitation from 2 independent batches of purified enzymes.

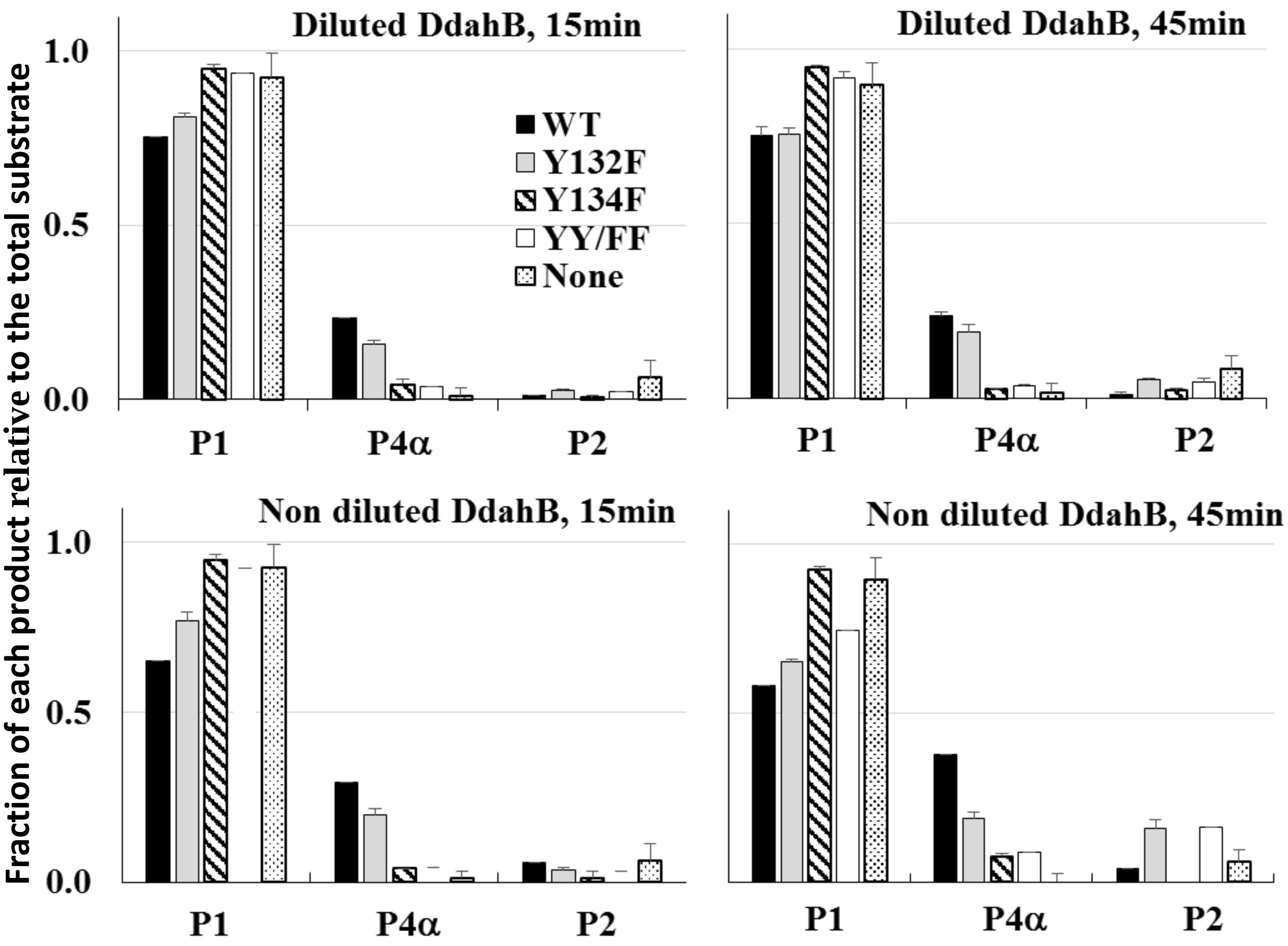

Figure S7: Superimposition of the MlghC (orange) and DdahC (blue) monomers.

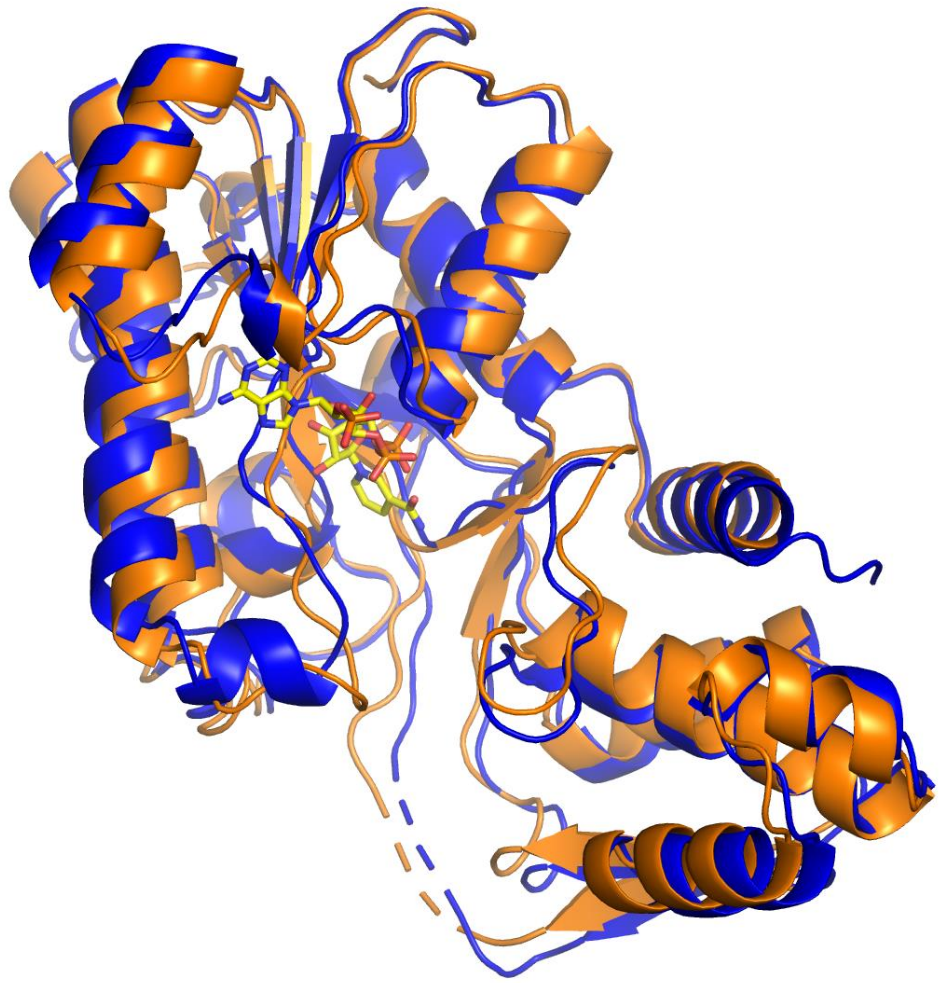

Figure S8: Superposition of MlghC (orange) with human GDP-L-fucose synthase (GFS) (grey). NADP in MlghC is coloured as in Figure 10A. NADP and GDP-L-fucose molecules found in GFS have their carbons coloured purple (PDB 4bl5).

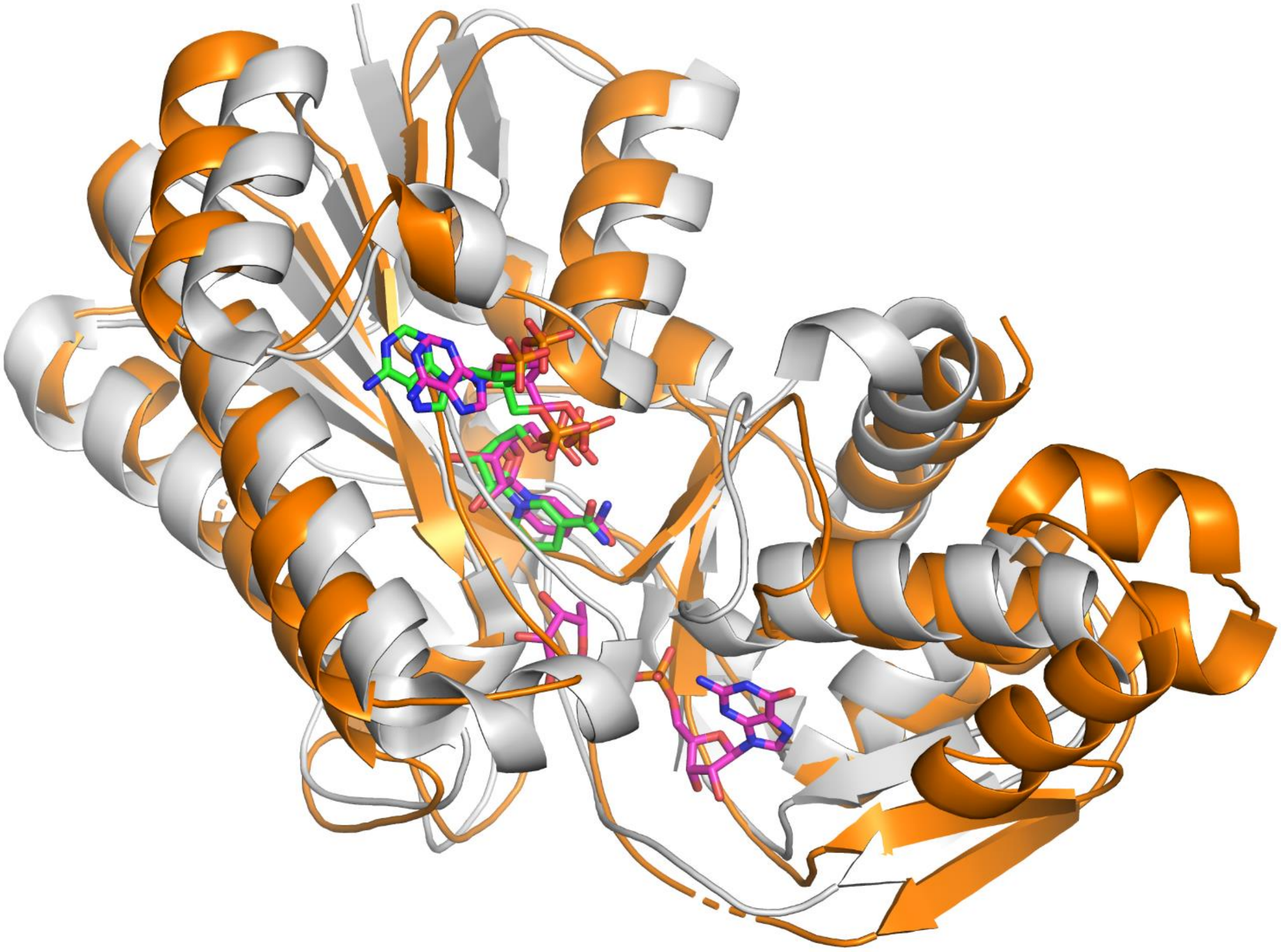

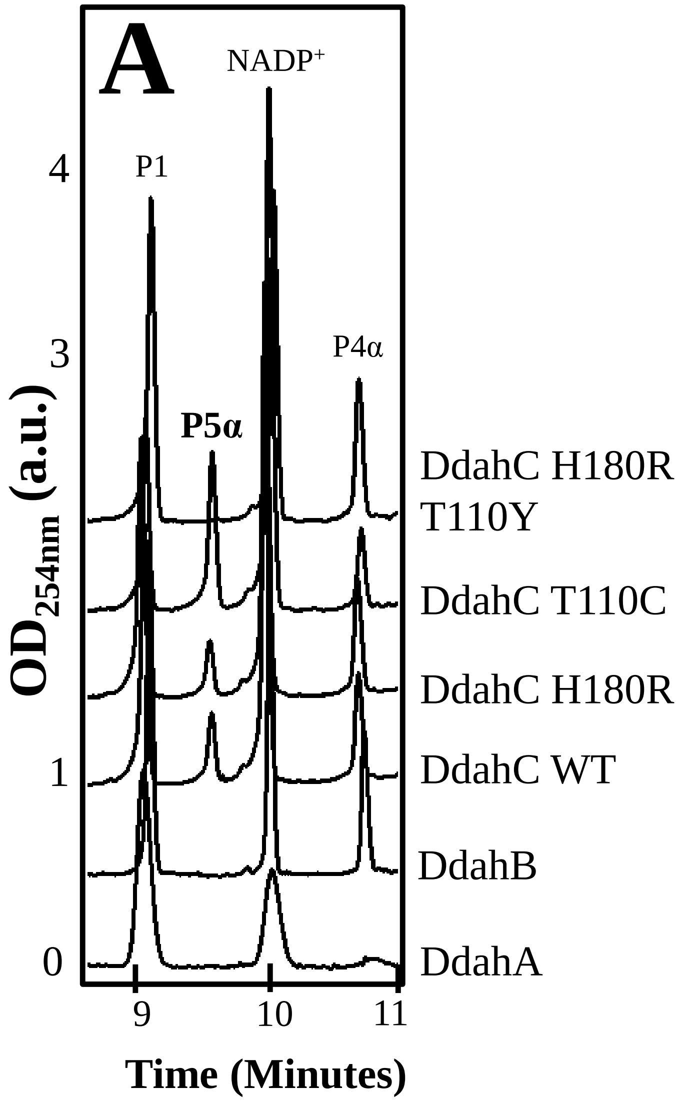

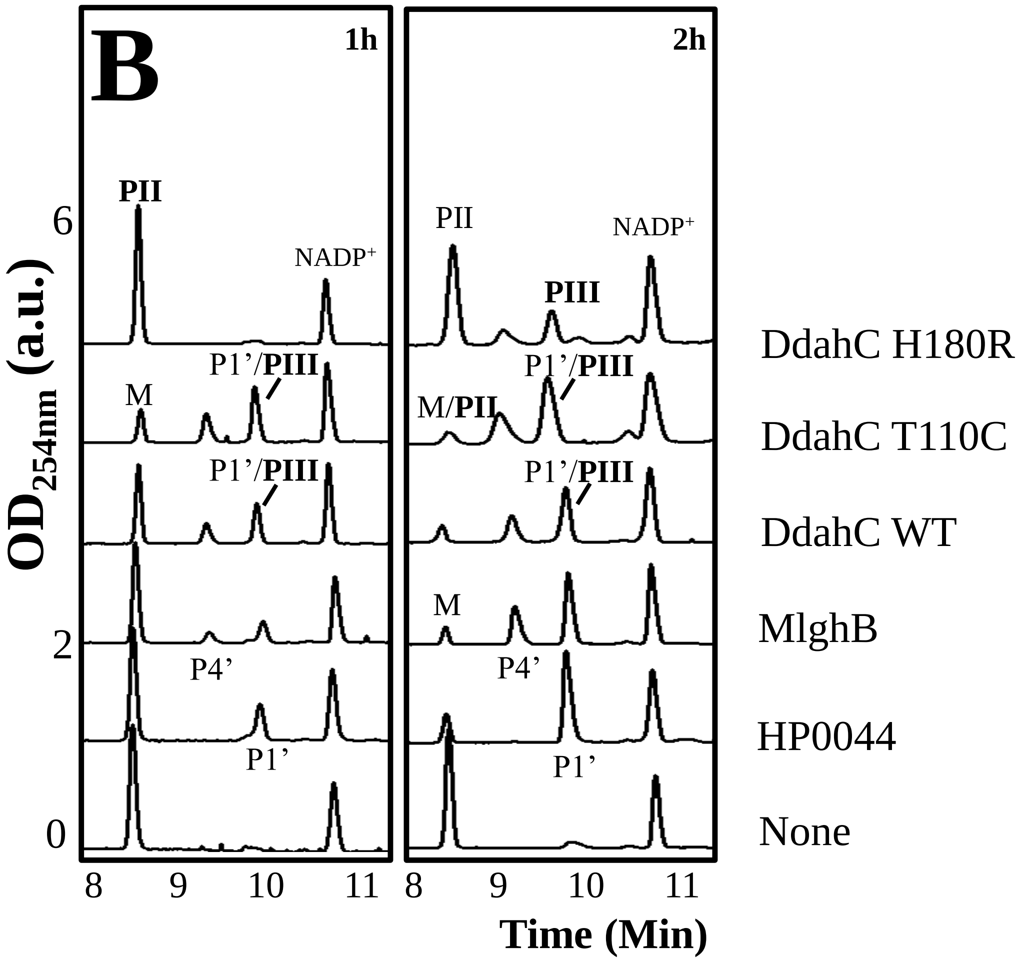

Figure S9: Catalytic activity of mutated DdahC reductase. Panel A. CE profile at 45 min for reductase activity on heptose whereby a single substrate P4α was generated by 1.5 µM DdahA and 0.4 µM DdahB. Reactions contained 0.3 mM of heptose, 0.65 mM of NADPH/$^+$ and 0.2 µM of reductase in 11.5 µl. **Panel B.** Time course of activity on GDP-mannose whereby the substrate P4’ was generated by 0.27 µM HP0044 and 0.4 µM MlghB using 0.15 mM of mannose, 0.3 mM of NADPH/$^+$ and 0.2 µM of reductase in 10 µl.

Figure S10: Representative examples of Coomassie-stained SDS-PAGE gels for purified proteins diluted to equal concentrations for use in kinetic analyses. Panel A. Epimerases. **Panel B.** Reductases. For each mutated enzyme, concomitant expression and purification of wild-type enzyme was performed so that aged-matches enzymes diluted to the same concentration could be used to compare catalytic efficacy. Concentrations were determined by Bradford and concentration matched preparations were then run on SDS-PAGE gels. Each mutation was tested out of 2-3 independent expressions and purifications. The additional band seen in MlghB Y132F/Y134F was consistently observed in 2 independent preparations and could not be eliminated. Since this mutant was inactive like its pure Y134F counterpart, this was not pursued further.

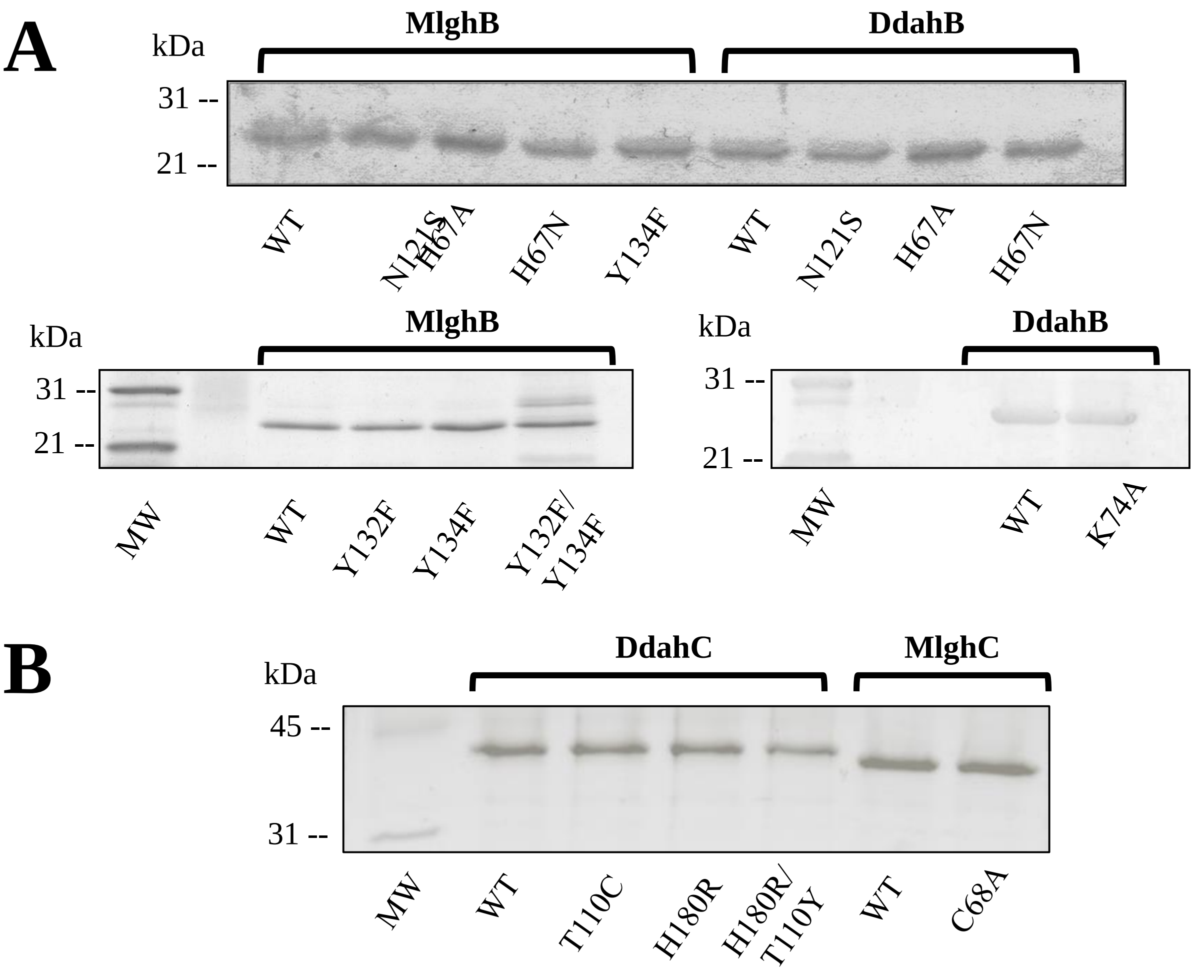

Supplement: Supplemental Figures S1–S10 and Tables S1–S7 [file mmc1.pdf]
